# High-dose dexmedetomidine versus dexamethasone supplementation to interscalene brachial plexus block in arthroscopic shoulder surgery: A randomized, controlled trial

**DOI:** 10.1371/journal.pone.0353121

**Published:** 2026-08-07

**Authors:** Thirada Srinil, Busara Sirivanasandha, Pathom Halilamien, Pawinee Pangthipampai, Jerattikul Phumchaitheerachort, Patcharaon Sangjak, Suppachai Poolsuppasit

**Affiliations:** Department of Anesthesiology, Faculty of Medicine Siriraj Hospital, Mahidol University, Bangkok, Thailand; PLOS: Public Library of Science, UNITED KINGDOM OF GREAT BRITAIN AND NORTHERN IRELAND

## Abstract

**Background:**

In this double-blind, randomized controlled trial, we compared the efficacy of perineural 2 µg/kg dexmedetomidine and 5 mg dexamethasone as adjuvants to bupivacaine for postoperative pain control in patients undergoing arthroscopic shoulder surgery. We hypothesized that the combination of bupivacaine with dexmedetomidine would prolong analgesia compared with dexamethasone.

**Methods:**

One hundred twelve patients scheduled for arthroscopic shoulder surgery were randomized to receive 15 mL of 0.33% bupivacaine with either 2 µg/kg of dexmedetomidine or 5 mg of dexamethasone during ultrasound-guided interscalene brachial plexus block (ISB). The primary outcome was the duration of analgesia (DOA), analyzed as a time-to-event variable using Kaplan–Meier survival curves with log-rank testing. Secondary outcomes included the duration of sensory block (DOS) and motor block (DOM), pain scores, incidence and severity of rebound pain, opioid consumption, patient satisfaction, and adverse effects. Longitudinal pain scores were evaluated using a regression model.

**Results:**

Of 112 randomized patients, 107 were included in the primary analysis. Dexmedetomidine and dexamethasone produced a similar DOA (median, 16.77 vs 12.48 h; median difference in location 5.24 h, 95% CI −0.47 to 9.17; p = 0.655). The dexmedetomidine group had a significantly longer DOS (19.8 vs. 17.3 h; mean difference 2.45 h, 95% CI 0.23 to 4.65; p = 0.031) and DOM (19.9 vs. 17.8 h; median difference in location 2.23 h, 95% CI 1.02 to 3.60; p = 0.008). Postoperative pain score at 6, 12, 18, and 24 hours were comparable, as were rebound pain, postoperative nausea and vomiting (PONV), patient satisfaction, and opioid consumption. The dexmedetomidine group had a higher incidence of intraoperative hypotension (risk difference [RD] 44.4%, 95% CI 30.0% to 59.9%; p < 0.001), bradycardia in the block room (RD 17.9%; p = 0.010) and intraoperatively (RD 27.6%; p = 0.004), and postoperative sedation (RD 10.9%; p = 0.027); however, all hemodynamic events were transient and did not require intensive care admission.

**Conclusions:**

Perineural dexmedetomidine (2 µg/kg) was not shown to be superior to dexamethasone (5 mg) in prolonging analgesia when added to bupivacaine for ISB in arthroscopic shoulder surgery. Although dexmedetomidine prolonged sensory and motor block duration, this did not translate into a longer DOA and was associated with higher rates of hemodynamic instability and sedation. These findings suggest limited clinical benefit and raise safety concerns regarding higher dexmedetomidine doses.

## Introduction

Interscalene brachial plexus block (ISB) targeting cervical nerve roots C5 to C7 is frequently performed to provide safe and effective postoperative analgesia for shoulder surgery, which commonly involves moderate to severe postoperative pain [1]. Continuous peripheral nerve block catheters offer the advantage of prolonged analgesia; however, improper execution of this technically demanding technique, combined with inadequate postoperative care team preparation, can lead to catheter dislodgement and suboptimal analgesia. As an alternative, single-shot ISB provides a simpler approach that delivers effective analgesia for a limited duration.

To extend the duration of postoperative analgesia, long-acting local anesthetics are often combined with adjuvants. Dexamethasone, a corticosteroid, has consistently been shown in systematic reviews to significantly prolong the duration of analgesia (DOA) when combined with local anesthetics [2,3]. It exerts analgesic effects by inhibiting phospholipase A2 and stimulating the glucocorticoid system, producing systemic anti-inflammatory effects. It is also thought to prolong the DOA by acting on nociceptive C fibers and inhibiting potassium channel function, thereby preventing nerve cell depolarization and the transmission of pain signals to the brain [4]. Perineural administration produces longer-lasting effects than the systemic route [5–7]. A systematic review identified 4 mg as a ceiling dose for perineural dexamethasone, extending analgesia by an average of 8 hours when combined with long-acting local anesthetics [8]. Despite these benefits, dexamethasone is generally avoided in patients with poorly controlled diabetes because of its hyperglycemic effects.

Alpha-2 adrenergic agonists such as clonidine and dexmedetomidine have also been shown to prolong the effects of brachial plexus block [9–11]. Of these, dexmedetomidine is more effective than clonidine in enhancing the analgesic effects of local anesthetics owing to its stimulation of hyperpolarization-activated cyclic nucleotide-gated channels, which prevents pain signal transmission, and its vasoconstriction of neural blood vessels [12–15]. Dexmedetomidine is increasingly used to enhance brachial plexus nerve block analgesia, with efficacy dependent on the route of administration; both intravenous (IV) and perineural routes significantly enhance pain relief [16,17].

Recent meta-analyses comparing these two adjuvants have reported conflicting findings, with some suggesting that dexamethasone is equivalent or superior to dexmedetomidine in prolonging analgesia [18–21]. Notably, dexmedetomidine doses in these studies ranged from 50–100 µg or 0.75–1 µg/kg. A prior dose-finding study of ISB for arthroscopic shoulder surgery compared perineural dexmedetomidine at 1, 1.5, and 2 µg/kg and found that 2 µg/kg produced the longest DOA, although it was also associated with a higher incidence of intraoperative hypotension [22].

Because pain, sensory, and motor fibers differ in size and myelination, they exhibit different sensitivities to local anesthetics [23]. Peripheral nerve block efficacy is therefore commonly characterized by the DOA (time from block placement to first pain or rescue analgesia) and the durations of sensory and motor block (DOS and DOM; time from block placement to recovery of sensation and motor strength, respectively) [24,25], allowing differentiation between prolonged analgesia and prolonged sensory or motor block.

As no study had compared 2 µg/kg perineural dexmedetomidine with 5 mg perineural dexamethasone when combined with bupivacaine, this trial aimed to address this gap. We selected 2 µg/kg dexmedetomidine, calculated from ideal body weight, based on the dose-finding evidence above [22], to standardize drug exposure across patients and to evaluate whether this higher dose—beyond the range previously compared with dexamethasone—would provide additional clinical benefit. However, because this dose exceeds those typically studied, the potential for prolonged analgesia needed to be weighed against the risk of dose-dependent adverse effects, including bradycardia, hypotension, and sedation. The primary outcome was DOA. We hypothesized that combining bupivacaine with dexmedetomidine at 2 µg/kg would result in a longer DOA compared with dexamethasone at 5 mg. Secondary outcomes included DOS, DOM, pain scores, incidence and severity of rebound pain, opioid consumption, patient satisfaction, and adverse effects.

## Materials and methods

This double-blinded, randomized controlled trial was approved by the Siriraj Institutional Review Board (COA Si 256/2020) on April 1, 2020, and conducted at Siriraj Hospital, Bangkok, Thailand, from December 9, 2020, to October 26, 2022. Patients aged 18–80 years undergoing arthroscopic shoulder surgery were enrolled. Exclusion criteria included American Society of Anesthesiologists (ASA) physical status greater than 3, body mass index (BMI) greater than 35 kg/m², severe cardiovascular or respiratory disorders, nerve damage at the operative site, allergies or contraindications to study drugs, coagulopathy, septic arthritis, pregnancy, or inability to self-assess pain. Written informed consent was obtained from each participant before enrollment. Patients who withdrew consent, had a failed ISB, or required conversion to open arthrotomy met the study’s withdrawal criteria.

The study was carried out in compliance with the International Council for Harmonization Good Clinical Practice guidelines, the Belmont Report, and the Declaration of Helsinki. The trial was registered at ThaiClinicalTrials.org on April 9, 2020 (https://www.thaiclinicaltrials.org/show/TCTR20200409002). The full trial protocol, including the statistical analysis plan, is accessible via the trial registration entry at ThaiClinicalTrials.org (TCTR20200409002). No separate protocol publication was prepared.

There were no important changes to the trial methods or outcomes after trial commencement. The trial was terminated after reaching the pre-specified target sample size; no interim analyses were planned or conducted.

Patients and the public were not involved in the design, conduct, or reporting of this trial.

Patients were randomized 1:1 to receive dexamethasone (5 mg) or dexmedetomidine (2 µg/kg, based on ideal body weight, calculated as height [cm] – x, where x = 100 for males and x = 105 for females) by the principal investigator (S.P.) using a computer-generated sequence (www.randomizer.org), with assignments sealed to prevent selection bias. A blinded research assistant prepared the study drug by mixing it with 5 mL of saline in a 5-mL syringe, then combined it with 10 mL of 0.5% bupivacaine. The patient, anesthesiologist, and research coordinator remained blinded to group assignments. Both study solutions were clear and colorless and were prepared in identical syringes of the same total volume (15 mL), rendering them visually indistinguishable.

Patients were instructed to fast after midnight and receive maintenance IV fluids (glucose- or non-glucose-containing) after providing informed consent. No analgesic premedication was given; patients’ usual chronic analgesic were continued when applicable. Patients received standard preoperative care and were then transferred to the regional block room, where monitoring was established, including continuous pulse oximetry (SpO_2_), continuous electrocardiography (ECG), and non-invasive blood pressure (NIBP) measured at 5-minute intervals. Supplemental oxygen was administered via nasal cannula at 3 L/min. All patients received fentanyl 25–50 µg IV to alleviate anxiety.

Patients underwent an ISB targeting the C5 and C6 nerve roots. All blocks were performed or directly supervised by attending anesthesiologists experienced in ultrasound-guided regional anesthesia. The skin was sterilized with chlorhexidine and infiltrated with 2% lidocaine. A sterile dressing (Tegaderm^™^, 3M Health Care, USA) covered the linear array transducer, which was placed in the transverse plane over the interscalene groove to visualize the carotid artery and nerve roots of the brachial plexus. An ultrasound-guided 50 mm needle (Stimuplex^®^ Ultra 360, B. Braun, Germany) was inserted in plane until the tip was adjacent to the C5 and C6 nerve roots. After confirming the absence of blood on aspiration, 15 mL of the solution was injected lateral to the brachial plexus sheath, separating the middle scalene muscle laterally (extrafascial injection technique).

Sensory function was assessed using a pinprick test at the C5 (deltoid insertion) and C6 (thenar eminence) dermatomes, scored from 0 (loss of sensation) to 2 (normal sensation). Muscle strength was tested at C5 (shoulder abduction) and C6 (wrist extension), scored from 0 (no resistance) to 2 (full resistance). Assessments were performed every 5 minutes, three times, to determine nerve blockade onset. Sedation was evaluated at 15 minutes using a 4-point scale ranging from 0 to 3: 0 (awake and alert), 1 (drowsy but easily arousable), 2 (somnolent and mostly asleep), and 3 (arousable only with significant physical stimulation or unarousable). Patients with inadequate anesthesia were to be excluded from the trial and receive appropriate pain management.

Standard monitoring was performed before inducing general anesthesia with endotracheal intubation. Intraoperative anesthetic agents included: induction agents (fentanyl [25–50 µg], propofol [1–2 mg/kg] and cisatracurium [0.15–0.2 mg/kg]); maintenance agents (N_2_O, O_2_, desflurane [1 minimum alveolar concentration (MAC)], and fentanyl [25–50 µg] when systolic blood pressure (SBP) exceeded 120% of baseline); reversal agents (neostigmine [2.5 mg] with atropine [1.2 mg] or glycopyrrolate [0.4 mg]), and antiemetic prophylaxis (ondansetron [8 mg]).

Hypotension was defined as SBP less than 80% of baseline, and bradycardia was defined as heart rate less than 50 beats per minute. Heart rate was monitored continuously via ECG, and blood pressure was measured by NIBP at 5-minute intervals throughout the block room and intraoperative periods. Management of hypotension and bradycardia was at the discretion of the attending anesthesiologist.

Patients were observed in the post-anesthesia care unit (PACU) for 1–2 hours. Sedation and pain were assessed using the sedation scale and the 0–10 numerical rating scale (NRS), respectively. Morphine patient-controlled analgesia (PCA) (Sapphire™ Multi-Therapy Infusion Pump, Eitan Medical, Israel) was provided for pain relief (1 mg/bolus, 5-minute lockout, 30 mg/4-hour limit for 30 hours). PCA was initiated when the NRS exceeded 4 and repeated if pain persisted after 5 minutes. Postoperative analgesia included acetaminophen (500 mg, two tablets every 6 hours) and etoricoxib (90 mg, daily) for 3 days.

Patients self-reported initial surgical pain and PCA usage via a questionnaire for 30 hours, along with the return of normal sensation and muscle strength. A research assistant reinforced proper use of the self-report questionnaire and IV PCA throughout the ward, waiting room, block room, and PACU on the day of surgery and the following day.

The primary outcome was the DOA, defined as the time from local anesthetic injection to the first report of pain or first PCA activation, whichever occurred first. Secondary outcomes included the DOS and DOM—measured from injection to return of normal hand sensation and muscle strength, respectively—total intraoperative fentanyl consumption (µg/kg/h), postoperative morphine consumption (mg), NRS pain scores at 0, 6, 12, 18, and 24 hours, incidence and severity of rebound pain (NRS ≥ 7 within 12 hours after sensory recovery; severity defined as the difference between maximum pain after recovery and minimum pain before block resolution), adverse effects (hypotension and bradycardia in the block room and intraoperatively, postoperative nausea and vomiting [PONV] in the PACU and on the ward, and sedation score >0 at 60 minutes), and patient satisfaction (0–100 scale).

### Sample size and power calculation

Previous studies reported that groups receiving 5 mg of dexamethasone or 2 µg/kg of dexmedetomidine achieved a mean DOA ± standard deviation (SD) of 18 ± 1.95 and 20.4 ± 3.96 hours, respectively [7,22]. A superiority trial was designed using an automated sample size calculator, with statistical significance set at a two-sided alpha of 0.05 (type I error = 5%) and 80% power (type II error = 20%); each group required a sample size of 50 patients. Accounting for approximately a 10% dropout rate, each group was set to consist of 56 patients, for a total of 112.

### Statistical analysis

Outcome measures were classified as time-to-event, continuous, or categorical variables.

The primary outcome, DOA (hours), was analyzed as a time-to-event variable using Kaplan–Meier survival curves, with between-group comparison using the log-rank test. Median (interquartile range [IQR]) DOA and the corresponding median difference in location with 95% confidence intervals (CIs) were reported for descriptive and comparative purposes.

Continuous secondary outcomes included DOS and DOM (hours), intraoperative fentanyl consumption (µg/kg/h), postoperative morphine consumption (mg), NRS pain scores (0–10) at 0, 6, 12, 18, and 24 hours, rebound pain severity (NRS difference), and patient satisfaction scores (0–100). Normality was assessed with the Kolmogorov–Smirnov test. Normally distributed variables were summarized as mean ± SD, and between-group comparisons were performed using the independent-samples t test; group differences were expressed as mean differences (MDs) with 95% CIs derived from IBM SPSS Statistics. Non-normally distributed variables were presented as median (IQR) and compared with the Mann–Whitney U test. To provide a clinically interpretable estimate of effect size, the between-group difference was additionally expressed as the Brown–Mood median difference in location with 95% CIs, obtained using the coin package in R. Longitudinal NRS scores were further evaluated using a regression model including time, treatment group, and their interaction, with adjusted coefficients and 95% CIs reported from marginal estimates.

Categorical secondary outcomes—rebound pain incidence (yes/no), hypotension in the block room, bradycardia in the block room, intraoperative hypotension, intraoperative bradycardia, PONV in the PACU, PONV on the ward, sedation score >0 at 60 minutes (yes/no), and other adverse events—were summarized as frequencies (percentages). Between-group comparisons were made using the chi-square test or Fisher’s exact test, as appropriate, and the effect size was reported as risk differences (RDs) with 95% CIs, estimated in R using the fmsb package.

All analyses were conducted on a complete-case basis; only participants with available data for a given outcome were included in the corresponding analysis. The reported analyses were performed in the evaluable population after exclusion of patients with major post-randomisation protocol deviations considered likely to confound pain-related outcomes, specifically receipt of non-study analgesics on the day of surgery. Patients who required conversion to open arthrotomy were withdrawn according to the prespecified withdrawal criteria. No imputation of missing values was performed. For the Kaplan–Meier analysis of DOA, patients who did not report pain or request rescue analgesia during the observation period were censored at the end of follow-up.

All tests were two-sided with a significance level of 0.05. Primary and secondary analyses and calculation of MDs with 95% CIs were performed using IBM SPSS Statistics, version 26.0 (IBM Corp., Armonk, NY, USA). Brown–Mood median differences and risk differences with 95% CIs were obtained using R (R Foundation for Statistical Computing, Vienna, Austria) in RStudio (version 2023.09.1; Posit Software, PBC), with the coin package for the Brown–Mood median test and the fmsb package for risk-difference estimation.

## Results

**Fig 1:**
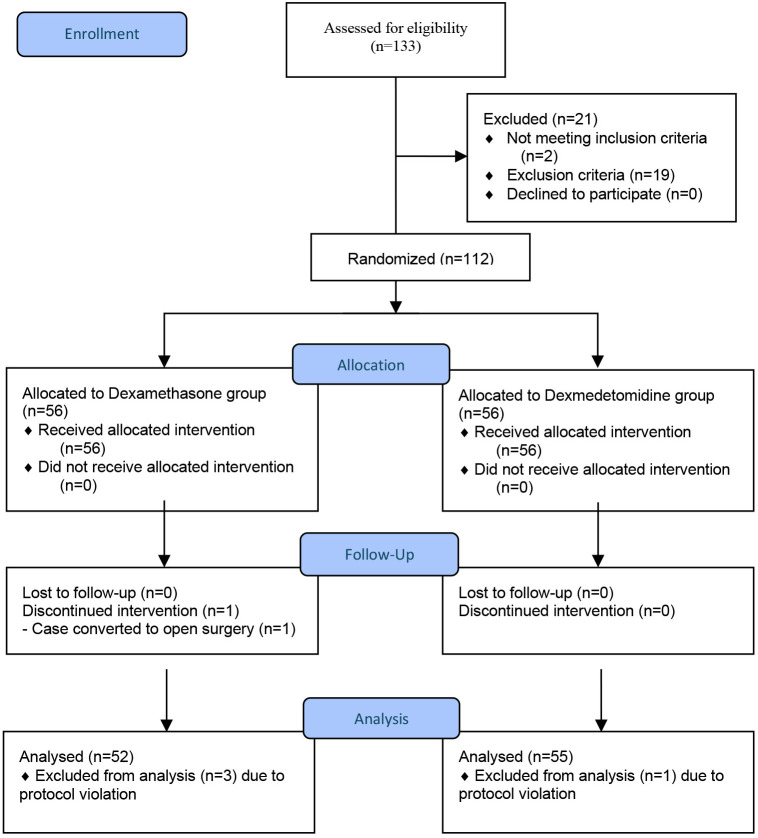
CONSORT patient flow diagram.

Of 133 patients evaluated for eligibility, 2 did not meet the inclusion criteria, and 19 meeting exclusion criteria were excluded. The remaining 112 patients were randomized 1:1 to the dexamethasone (n = 56) or dexmedetomidine (n = 56) group. Three surgeons with comparable experience participated in the study, and four attending anesthesiologists performed the ISB directly or through a supervised anesthesiology resident and fellow. All ISBs were successful and provided adequate anesthesia; no patients were excluded because of failed ISB. One patient in the dexamethasone group was withdrawn because arthroscopic surgery was converted to open surgery, according to the prespecified withdrawal criteria. Three additional patients in the dexamethasone group and one in the dexmedetomidine group were excluded from all analyses because postoperative administration of non-study analgesics on the day of surgery was considered likely to confound postoperative pain-related outcomes (Fig 1).

Baseline demographic and clinical characteristics—including age, sex, height, weight, BMI, ASA physical status, comorbidities, preoperative pain NRS, diagnosis, surgical laterality, type of surgery, surgeon, and duration of surgery—were comparable between groups (Table 1).

**Table 1:** Baseline demographic data, baseline pain NRS, block time, and operative time of patients receiving dexmedetomidine or dexamethasone as ISB adjuvants (n = 107).

Characteristics	Dexmedetomidine (n = 55)	Dexamethasone (n = 52)	p-value
Age (year)	63.0 [55-69]	62.5 [52.3-69]	0.963
Gender			0.062
Male	30 (54.5)	19 (36.5)	
Female	25 (45.5)	33 (63.5)	
Weight (kg)	65.0 [58-76]	61.5 [56.7-72.9]	0.209
Height (cm)	165.0 [155-170]	160.0 [155-166.8]	0.110
BMI (kg/ m^2^)	25.1 ± 3.2	25 ± 3.7	0.966
Operation			0.971
RC repair	45 (81.8)	41 (78.8)	
Labral repair	5 (9.1)	5 (9.6)	
Frozen shoulder surgery	3 (5.5)	4 (7.7)	
Other arthroscopic shoulder surgery (%)	2 (3.6)	2 (3.8)	
Laterality (right)	37 (67.3)	36 (69.2)	0.828
American Society of Anesthesiologist classification			1.000
1	13 (23.6)	12 (23.1)	
2	37 (67.3)	36 (69.2)	
3	5 (9.1)	4 (7.7)	
Coexisting diseases			
Hypertension	23 (41.8)	22 (42.3)	0.959
Dyslipidemia	24 (43.6)	18 (34.6)	0.340
OSA	4 (7.3)	2 (3.8)	0.679
DM	9 (16.4)	8 (15.4)	0.890
MDD	0 (0)	1 (1.9)	0.486
Pain NRS at rest (0–10)	0 [0-4]	0 [0-3]	0.576
Pain NRS on movement (0–10)	5 [0-7]	4 [0-5.8]	0.236
Block time (minutes)	3 [3 –4]	4 [3 –5]	0.110
Operative time (minutes)	105.1 ± 39.1	106.7 ± 32.5	0.821
Initiation of ISB to start operation interval (minutes)	55 [48-75]	55 [47-72]	0.767
Surgeon			0.157
1	46 (83.6)	35 (67.3)	
2	8 (14.5)	15 (28.8)	
3	1 (1.8)	2 (3.8)	

Values are expressed as medians [interquartile range] or numbers (percentages), except BMI and operative time, which are expressed as mean ± standard deviation. Abbreviations: body mass index = BMI; diabetes mellitus = DM; major depressive disorder = MDD; numerical rating scale = NRS; obstructive sleep apnea = OSA; rotator cuff = RC. P < 0.05 considered statistically significant.

**Fig 2:**
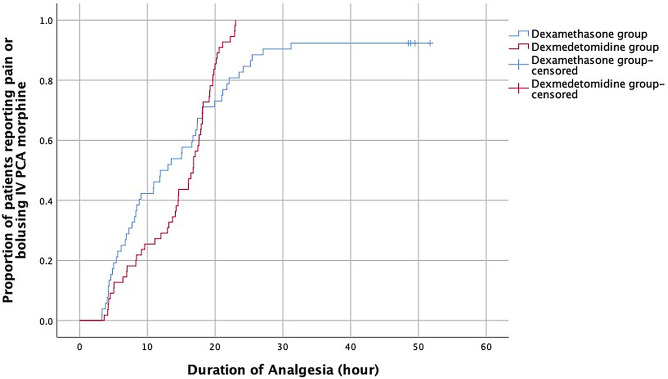
Kaplan-Meier survival plot for duration of analgesia. Duration of analgesia was plotted against the proportion of pain presence or patient-administered morphine in each arm. The dexamethasone group is presented in blue and the dexmedetomidine group in red. In the dexamethasone group, four patients were censored due to the absence of both pain and the request for rescue analgesia. Abbreviations: IV, intravenous; PCA, patient-controlled analgesia.

The median DOA appeared longer in the dexmedetomidine group than in the dexamethasone group (16.77 [9.62–19.2] vs. 12.48 [6.28–21.09] h; median difference in location 5.24 h, 95% CI −0.47 to 9.17; p = 0.655); however, this difference was not statistically significant. The Kaplan-Meier curve displays the time to the first IV PCA morphine bolus or first reported postoperative pain during admission. Four patients in the dexamethasone group reported no pain or requested rescue analgesia during admission (Fig 2). The distributions of DOA, DOS, and DOM by treatment group are shown in S2 Fig.

Dexmedetomidine, compared with dexamethasone, was associated with significantly longer DOS (MD 2.45 h, 95% CI 0.23 to 4.65; p = 0.031) and DOM (median difference in location 2.23 h, 95% CI 1.02 to 3.60; p = 0.008) (Table 2).

**Table 2:** Comparison of the duration of analgesic, sensory and motor block, perioperative opioid consumption, patient satisfaction, and adverse effects between groups (n = 107).

Characteristics	Dexmedetomidine (n = 55)	Dexamethasone (n = 52)	Effect estimate (95% CI)	p-value
Duration of analgesia (hours)	16.77 [9.62-19.2]	12.48 [6.28-21.09]	5.24 (−0.47 to 9.17)	0.655
Duration of sensory block (hours)	19.8 ± 5.34	17.3 ± 5.64	MD 2.45 (0.23 to 4.65)	0.031
Duration of motor block (hours)	19.9 [16.6-23.1]	17.8 [14.1-19.6]	2.23 (1.02 to 3.60)	0.008
Rebound pain	16 (29.1%)	13 (25%)	RD 3.6% (−13.3 to 20.5%)	0.678
Rebound pain severity	6.5 [3.25-8]	5 [4 –8]	1 (−4–3)	0.965
Intraoperative consumption of fentanyl (mcg/ kg/ h)	0.56 [0.39-0.78]	0.66 [0.49-0.91]	−0.10 (−0.17 to 0.04)	0.104
Postoperative morphine consumption (mg)	7 [4 –13]	5 [2 –13]	2 (−1–6)	0.324
Patient satisfaction (0–100)	100 [90-100]	100 [90-100]	20 (−0.01 to 20.00)	0.966
Adverse effects				
Hypotension in the block room	2 (3.6%)	2 (3.8%)	RD −0.2% (−7.4 to 7.0%)	1.000
Bradycardia in the block room	13 (23.6%)	3 (5.8%)	RD 17.9% (5.0 to 30.8%)	0.010
Intraoperative hypotension	53 (96.4%)	27 (51.9%)	RD 44.4% (30.0 to 59.9%)	< 0.001
Intraoperative bradycardia	30 (54.5%)	14 (26.9%)	RD 27.6% (9.8 to 45.5%)	0.004
PONV at PACU	1 (1.8%)	0 (0%)	RD 1.8% (−1.7 to 5.3%)	1.000
PONV in the ward	14 (25.5%)	7 (13.5%)	RD 12.0% (−2.8 to 26.8%)	0.118
Sedation score > 0 at 60 minutes	6 (10.9%)	0 (0%)	RD 10.9% (2.7 to 19.1%)	0.027

Values are expressed as medians [interquartile range] or numbers (percentages), except for duration of sensory block, which is expressed as mean ± standard deviation. Effect estimates are reported as the Brown–Mood median difference in location, mean differences (MDs), or risk differences (RDs), each with corresponding 95% confidence intervals, as appropriate. For skewed variables, the median difference in location is not necessarily equal to the arithmetic difference between the reported medians. Sedation score 0 indicates awake and alert; 1 indicates drowsy but easily arousable; 2 indicates somnolent and asleep most of the time; and 3 indicates unarousable or only arousable with significant physical stimulation. Abbreviations: NRS, numerical rating scale; PACU, postanesthetic care unit; PONV, postoperative nausea and vomiting. A p-value < 0.05 was considered statistically significant.

Both groups had similar intraoperative fentanyl consumption (p = 0.104). No significant differences in cumulative postoperative IV morphine consumption between the two groups were observed (p = 0.324) (Table 2).

Bradycardia was more frequent in the dexmedetomidine group both in the block room (RD 17.9%, 95% CI 5.0% to 30.8%; p = 0.010) and intraoperatively (RD 27.6%, 95% CI 9.8% to 45.5%; p = 0.004). Intraoperative hypotension was also more frequent with dexmedetomidine (RD 44.4%, 95% CI 30.0% to 59.9%; p < 0.001). One patient in the dexmedetomidine group required intraoperative continuous IV dopamine; the remaining patients received bolus ephedrine, atropine, or norepinephrine as clinically indicated. No patients experienced hypotension or bradycardia in the PACU or required ICU admission. At 60 minutes after surgery, the dexmedetomidine group had a significantly higher proportion of patients with sedation scores above 0 (p = 0.027). The PONV incidence was comparable between the two groups (Table 2).

**Fig 3:**
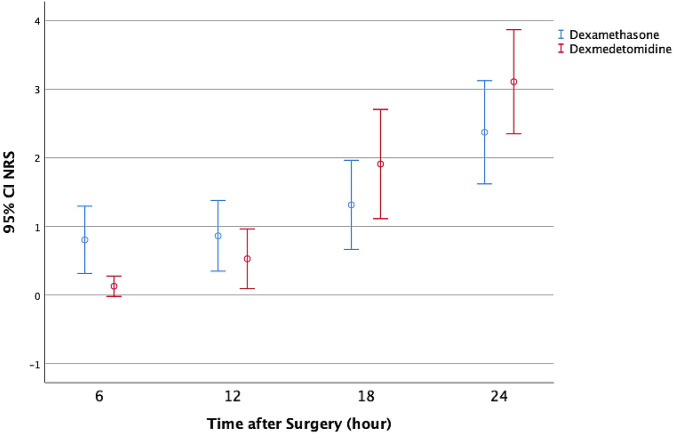
Adjusted pain predictions at 6, 12, 18, and 24 hours after surgery in patients receiving dexmedetomidine and dexamethasone. Postoperative pain, assessed using the numeric rating scale (NRS) ranging from 0 to 10, was evaluated at 6-hour intervals for each study arm. The data are presented with a linear-risk model adjusted value, along with its corresponding 95% confidence interval (CI). The dexamethasone group is represented in blue, while the dexmedetomidine group is depicted in red. The dexmedetomidine arm exhibited lower pain scores at 6 hours but higher scores at 24 hours compared to the dexamethasone group.

Postoperative pain scores did not differ significantly between groups at any time point, although the dexmedetomidine group had numerically lower scores at 6 hours and higher scores at 24 hours; neither difference was statistically significant (Table 3 and S1 Fig). In the dexmedetomidine group, pain scores (coefficient [95% CI]) increased significantly from hours 6–18 (1.78 [1.01, 2.56]; p < 0.001) and hours 6–24 (2.98 [2.13, 3.83]; p < 0.001); in the dexamethasone group, pain scores increased significantly only from hours 6–24 (1.57 [0.68, 2.45]; p = 0.001) (S1 Table). However, the linear regression model comparison of overall pain scores showed no significant differences (Fig 3).

**Table 3:** Comparison of pain NRS at 0, 6, 12, 18 and 24 hours after surgery in patients receiving dexmedetomidine or dexamethasone (n = 107).

Hours post-surgery	Pain NRS (0–10)		Coef (95% CI)	p-value
	Dexmedetomidine (n = 55)	Dexamethasone (n = 52)		
0 h	0 ± 0	0 ± 0	N/A	N/A
6 h	0.13 ± 0.546	0.8 ± 1.744	− 0.68 (- 1.40, 0.04)	0.066
12 h	0.53 ± 1.608	0.86 ± 1.833	− 0.34 (- 1.09, 0.42)	0.383
18 h	1.91 ± 2.946	1.31 ± 2.311	0.51 (- 0.25, 1.44)	0.169
24 h	3.11 ± 2.807	2.37 ± 2.676	1.57 (- 0.25, 1.72)	0.144

Values are expressed as mean ± SD. Abbreviations: Coef, coefficient; 95% CI, 95% confidence interval; N/A, not applicable; NRS, numerical rating scale. P < 0.05 considered statistically significant.

## Discussion

In this double-blind randomized trial, perineural dexmedetomidine (2 µg/kg) as an ISB adjuvant did not significantly prolong the DOA compared with dexamethasone 5 mg—the primary outcome—although it produced longer DOS and DOM and was associated with higher rates of sedation, bradycardia, and hypotension. Postoperative opioid consumption, pain scores, PONV, and patient satisfaction also remained comparable between groups, indicating that the dose escalation did not confer additional clinical benefit.

Several meta-analyses have indicated that dexamethasone is either superior or equivalent to dexmedetomidine in prolonging the DOA, using dexmedetomidine doses ranging from 50–100 µg or 0.75–1 µg/kg [18–21]. Although 2 µg/kg dexmedetomidine was selected based on prior dose-finding work in arthroscopic shoulder surgery [22], this regimen is higher than the fixed doses commonly used in previous trials. Our study is likely the first to compare this higher dose with dexamethasone, and we found no statistically significant difference in DOA. Although the dexmedetomidine group showed a numerically longer median DOA (16.77 vs 12.48 h), this difference did not reach significance. As this study was designed as a superiority trial, this finding should not be interpreted as evidence of equivalence. The observed difference of approximately 5 hours may be clinically relevant, but the confidence interval included zero, indicating uncertainty regarding the true magnitude of benefit. Moreover, a post hoc sample size calculation based on the observed means and variability (mean DOA 12.85 vs 14.71 h; pooled SD 7.33) indicated that 358 patients per group would be required to achieve 80% power, suggesting that the study was substantially underpowered for the primary outcome.

Prior dose-finding work [26] has identified a dose–response plateau for perineural dexmedetomidine with long-acting local anesthetics at approximately 30–50 µg, suggesting that our 2 µg/kg regimen likely exceeded the range that yields additional analgesic benefit while increasing dose-related hemodynamic effects. Moreover, the comparator dose of dexamethasone (5 mg) approaches the ceiling of its analgesia-prolonging effect, leaving little room for superiority. Consistent with these pharmacological considerations, we observed longer DOS and DOM with dexmedetomidine but no advantage in DOA. This discrepancy is also compatible with our time-to-event definition of DOA—time to first pain or first IV PCA activation, with censoring in patients who never triggered either event—and the fact that ISB does not cover all potential nociceptive inputs. Prolongation of block characteristics therefore does not necessarily translate into delayed analgesic demand. Collectively, these factors help explain why the primary endpoint did not demonstrate superiority despite pharmacological prolongation of DOS and DOM and a numerically longer DOA.

The pharmacological profiles of the two adjuvants further inform these findings. Perineural dexamethasone appears to act primarily via systemic absorption with glucocorticoid-mediated vasoconstrictive and anti-inflammatory effects, whereas perineural dexmedetomidine produces more pronounced local antinociception through ion-channel modulation and regional vasoconstriction, with additional systemic α_2_-adrenergic effects [13,19,27–30]. These mechanistic differences are consistent with the observed prolongation of DOS and DOM by dexmedetomidine without a corresponding extension of DOA.

In contrast to a previous study [16], the differential sensorimotor effect of dexmedetomidine—DOS extension without prolonged DOM—was not observed in our trial. However, DOS and DOM were based on patient self-reports, which may introduce inaccuracies. Regarding rebound pain, although recent studies have suggested that perineural dexamethasone may attenuate this phenomenon following peripheral nerve block [31,32], none included perineural dexmedetomidine as a comparator. Our study observed similar rebound pain rates and severity between groups.

Hemodynamic effects such as hypotension and bradycardia have been associated with dexmedetomidine in prior studies, though previous comparisons with dexamethasone at lower doses found no significant differences in incidence [33–35]. In our study, the higher dose of dexmedetomidine was associated with significantly greater rates of these effects, supporting the role of systemic absorption as suggested by previous research [36]. Although bradycardia and hypotension were more frequent in the dexmedetomidine group, these events were transient and manageable, as no patients had persistent hemodynamic instability in the PACU or required ICU admission. We also observed evidence of biphasic hemodynamic responses, characterized by immediate bradycardia onset in the block room followed by delayed hypotension. This pattern is consistent with the known pharmacokinetics of dexmedetomidine: initially high plasma concentrations activate alpha-2 receptors in vascular smooth muscle, causing peripheral vasoconstriction, hypertension, and reflex bradycardia; as plasma levels decrease, vasoconstriction subsides, and the combination of vasodilation, inhibition of sympathetic catecholamine release, and increased vagal activity results in delayed hypotension [29].

Dexmedetomidine also induces sedation through alpha-2 receptor binding at the locus coeruleus. Consistent with previous reports [4,34,36,37], sedation rates were higher in the dexmedetomidine group: 10.9% of patients were minimally sedated at 60 minutes after surgery compared with none in the dexamethasone group. Nevertheless, this did not delay PACU discharge. PONV incidence was similar between groups, consistent with comparable postoperative opioid consumption.

This study has several limitations. First, DOA, DOS, and DOM were based on patient self-reports without physical assessments of sensory and motor function recovery. Several participants reported recovery in the early morning, suggesting that blockade may have resolved during sleep before being reported. Second, the composite definition of DOA as time to first pain report or first IV PCA activation may have been influenced by patient-dependent factors, including individual pain perception, reporting behavior, and willingness to activate PCA. Third, the reported analyses were not based on a strict intention-to-treat principle, because patients with major post-randomisation protocol deviations were excluded from all analyses; however, these deviations were considered likely to invalidate interpretation of the pain-related outcomes. Fourth, ISB does not cover the supraclavicular nerve from the cervical plexus and may not cover the posterior arthroscopic port insertion site innervated by the C8 root. Although this could confound analgesia comparisons, one study found no significant differences between C5–C7 (conventional ISB) and C5–C8 root blocks in pain NRS or DOA [38], and there is no evidence of added benefit from an intermediate cervical plexus block over ISB alone; the latter remains the Grade A recommendation in the PROSPECT guidelines for rotator cuff repair surgery [39]. Fifth, dexamethasone-induced hyperglycemia was not investigated. Finally, outcomes may vary with different peripheral nerve blocks, local anesthetics, and surgical procedures, warranting further investigation.

In this randomized controlled trial, high-dose perineural dexmedetomidine (2 µg/kg) was not shown to be superior to dexamethasone (5 mg) in prolonging the DOA when used as an adjuvant to bupivacaine for ISB in arthroscopic shoulder surgery. Although dexmedetomidine significantly prolonged sensory and motor block duration, this did not translate into a meaningful clinical benefit and was associated with higher rates of hemodynamic instability and sedation. These findings suggest limited advantage and potential safety concerns with higher dexmedetomidine doses in this clinical setting.

## Supporting information

S1 Fig Pain at 0, 6, 12, 18, and 24 hours after surgery in patients receiving dexmedetomidine and dexamethasone. Postoperative pain, assessed using the numeric rating scale (NRS) ranging from 0 to 10, was evaluated at 6-hour intervals for each study arm, along with its corresponding 95% confidence interval (CI). The dexamethasone group was represented in blue, while the dexmedetomidine group was depicted in red. The dexmedetomidine arm exhibited lower pain scores at 6 hours but higher scores at 24 hours compared to the dexamethasone group.
(PDF)

S2 Fig Distribution of DOM, DOS, and DOA by treatment group. DOA: duration of analgesia; DOS: durations of sensory; DOM durations of motor.
(PDF)

S1 Table Margin test comparing pain NRS at 12, 18, and 24 hours with 6 hours after surgery in patients receiving dexmedetomidine or dexamethasone. (PDF)

S1 File Raw data. (XLSX)
