# Supplementary material for: High-dose dexmedetomidine versus dexamethasone supplementation to interscalene brachial plexus block in arthroscopic shoulder surgery: A randomized, controlled trial
Source: PLoS One. 2026 Aug 7;21(8):e0353121. doi: 10.1371/journal.pone.0353121 (PMC13450720; doi:10.1371/journal.pone.0353121)
Supplement: Supplementary file 1 [file pone.0353121.s001.pdf]

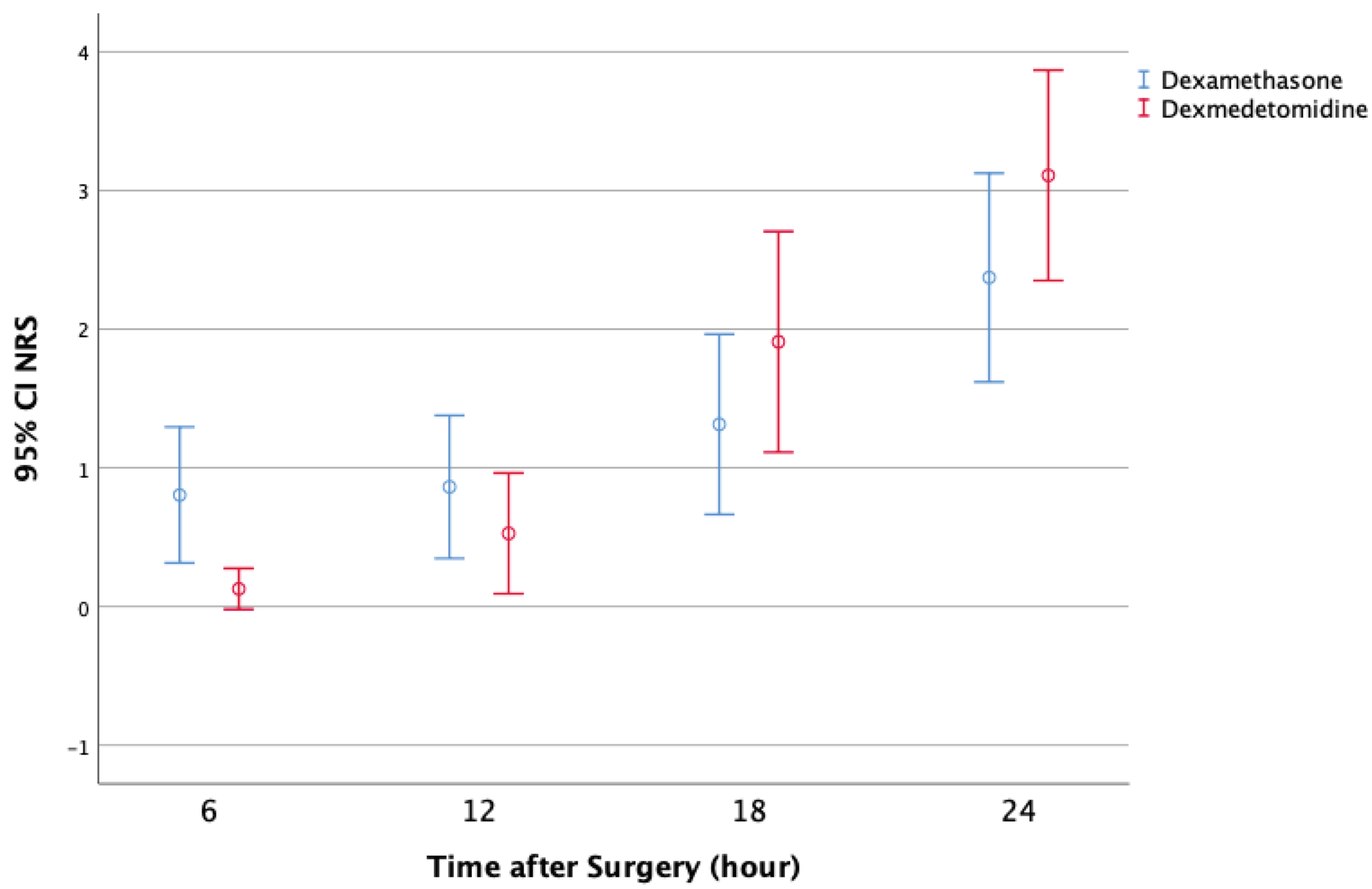

Supplementary Fig. S1. Pain at 0, 6, 12, 18, and 24 hours after surgery in patients receiving dexmedetomidine and dexamethasone. Postoperative pain, assessed using the numeric rating scale (NRS) ranging from 0 to 10, was evaluated at 6-hour intervals for each study arm, along with its corresponding 95% confidence interval (CI). The dexamethasone group was represented in blue, while the dexmedetomidine group was depicted in red. The dexmedetomidine arm exhibited lower pain scores at 6 hours but higher scores at 24 hours compared to the dexamethasone group.
