# Supplementary material for: High-dose dexmedetomidine versus dexamethasone supplementation to interscalene brachial plexus block in arthroscopic shoulder surgery: A randomized, controlled trial
Source: PLoS One. 2026 Aug 7;21(8):e0353121. doi: 10.1371/journal.pone.0353121 (PMC13450720; doi:10.1371/journal.pone.0353121)
Supplement: Supplementary file 2 [file pone.0353121.s002.pdf]

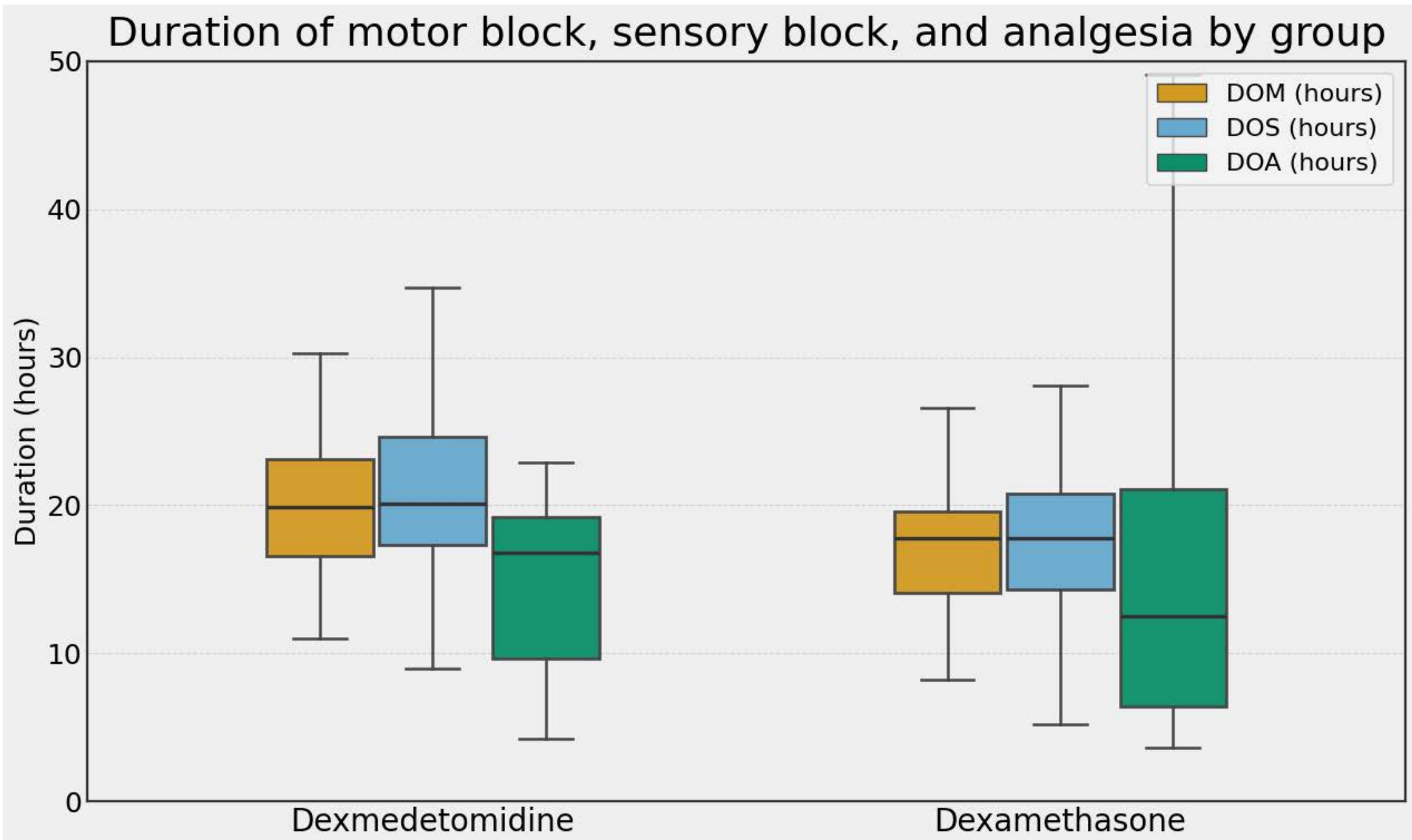

Supplementary Fig. S2. Distribution of DOM, DOS, and DOA by treatment group.

DOA: duration of analgesia; DOS: durations of sensory; DOM durations of motor.
