# Supplementary material for: High-dose dexmedetomidine versus dexamethasone supplementation to interscalene brachial plexus block in arthroscopic shoulder surgery: A randomized, controlled trial
Source: PLoS One. 2026 Aug 7;21(8):e0353121. doi: 10.1371/journal.pone.0353121 (PMC13450720; doi:10.1371/journal.pone.0353121)
Supplement: Supplementary file 3 [file pone.0353121.s003.pdf]

Supplementary Table S1. Margin test comparing pain NRS at 12, 18, and 24 hours with 6 hours after surgery in patients receiving dexmedetomidine or dexamethasone.

	**Dexmedetomidine**		**Dexamethasone**	
	Coef (95% CI)	p-value	Coef (95% CI)	p-value
12 h vs 6 h	0.4 (- 0.33, 1.13)	0.280	0.06 (- 0.69, 0.81)	0.878
18 h vs 6 h	1.78 (1.01, 2.56)	< 0.001	0.51 (- 0.29, 1.31)	0.214
24 h vs 6 h	2.98 (2.13, 3.83)	< 0.001	1.57 (0.68, 2.45)	0.001

Abbreviations: Coef = coefficient; 95% CI = 95% confidence interval; vs = versus.
