# Supplementary material for: High-dose dexmedetomidine versus dexamethasone supplementation to interscalene brachial plexus block in arthroscopic shoulder surgery: A randomized, controlled trial
Source: PLoS One. 2026 Aug 7;21(8):e0353121. doi: 10.1371/journal.pone.0353121 (PMC13450720; doi:10.1371/journal.pone.0353121)
Supplement: Supplementary file 5 [file pone.0353121.s007.pdf]

Peer Review Report

Manuscript Title

High-dose dexmedetomidine versus dexamethasone supplementation to interscalene brachial plexus block in arthroscopic shoulder surgery: a randomized, controlled trial

This randomized controlled trial addresses a clinically relevant question and offers a novel comparison between high-dose dexmedetomidine and dexamethasone as adjuvants to interscalene block. The study is well-structured; however, several important issues should be addressed:

Major Points

1. **Primary Outcome Interpretation**
   Despite a notable numerical difference in duration of analgesia (~5 hours), statistical significance was not achieved. This discrepancy should be clarified by discussing clinical versus statistical significance and specifying whether the study was powered for superiority, equivalence, or non-inferiority.
2. **Sample Size and Power**
   The assumed effect size differs from the observed outcome. A brief post hoc power analysis and discussion of potential underestimation of variability are recommended.
3. **Dexmedetomidine Dose Justification**
   The use of 2 μg/kg appears higher than the commonly recommended fixed doses. The rationale for weight-based dosing should be strengthened, with clearer discussion of dose-dependent risks versus benefits.
4. **Outcome Definition (DOA)**
   The composite definition (first pain or PCA activation) may introduce bias due to patient-dependent factors. This limitation should be acknowledged.
5. **Post-Randomization Exclusions**
   Excluding patients after randomization raises concerns regarding adherence to the intention-to-treat principle. Please clarify the analysis approach and consider including ITT-based results.
6. **Statistical Methods**
   Some methods (e.g., Brown–Mood test) appear unnecessarily complex. Simplification or clearer justification, along with inclusion of clinically interpretable effect sizes, is advised.
7. **Hemodynamic Adverse Events**
   The reported rates of hypotension and bradycardia are notably high. Definitions, monitoring protocols, and management strategies should be clearly described, along with a discussion of clinical safety implications.
8. **Data Availability Statement**
   Revise to comply with journal policy by stating that all relevant data are included within the manuscript and supporting files.

Minor Points:

- Correct typographical and grammatical errors.
- Ensure consistency in units and statistical reporting formats.
- Improve clarity and early definition of abbreviations.
- Revise tables (e.g., unclear satisfaction difference) and ensure complete labeling.
- Enhance figure clarity (e.g., Kaplan–Meier details).
- Shorten the AI disclosure section to align with journal standards.

Suggested Conclusion (Revised)

High-dose perineural dexmedetomidine (2 μg/kg) did not significantly prolong analgesia compared with dexamethasone (5 mg) as an adjuvant to bupivacaine in an interscalene block. Although it increased sensory and motor block duration, this did not translate into meaningful clinical benefit and was associated with higher rates of hemodynamic instability and sedation. These findings suggest limited advantage and potential safety concerns with higher dosing.
