# Supplementary material for: High-dose dexmedetomidine versus dexamethasone supplementation to interscalene brachial plexus block in arthroscopic shoulder surgery: A randomized, controlled trial
Source: PLoS One. 2026 Aug 7;21(8):e0353121. doi: 10.1371/journal.pone.0353121 (PMC13450720; doi:10.1371/journal.pone.0353121)
Supplement: Supplementary file 6 [file pone.0353121.s006.pdf]

Manuscript ID: PONE-D-25-27662
Title: High-dose dexmedetomidine versus dexamethasone supplementation to interscalene brachial plexus block in arthroscopic shoulder surgery: a randomized, controlled trial
Journal: PLOS ONE
Corresponding author: Suppachai Poolsuppasit

Reviewers' comments:

Reviewer's Responses to Questions
Comments to the Author

1. Is the manuscript technically sound, and do the data support the conclusions?

The manuscript must describe a technically sound piece of scientific research with data that supports the conclusions. Experiments must have been conducted rigorously, with appropriate controls, replication, and sample sizes. The conclusions must be drawn appropriately based on the data presented.
Reviewer #1: Yes
Reviewer #2: Yes
Reviewer #3: Yes
Reviewer #4: Partly

---

2. Has the statistical analysis been performed appropriately and rigorously?
Reviewer #1: Yes
Reviewer #2: Yes
Reviewer #3: Yes
Reviewer #4: I Don't Know

---

3. Have the authors made all data underlying the findings in their manuscript fully available?

The PLOS Data policy requires authors to make all data underlying the findings described in their manuscript fully available without restriction, with rare exception (please refer to the Data Availability Statement in the manuscript PDF file). The data should be provided as part of the manuscript or its supporting information, or deposited to a public repository. For example, in addition to summary statistics, the data points behind means, medians and variance measures should be available. If there are restrictions on publicly sharing data—e.g. participant privacy or use of data from a third party—those must be specified.
Reviewer #1: Yes
Reviewer #2: Yes
Reviewer #3: Yes
Reviewer #4: Yes

---

4. Is the manuscript presented in an intelligible fashion and written in standard English?

PLOS ONE does not copyedit accepted manuscripts, so the language in submitted articles must be clear, correct, and unambiguous. Any typographical or grammatical errors should be corrected at revision, so please note any specific errors here.
Reviewer #1: Yes
Reviewer #2: Yes
Reviewer #3: No
Reviewer #4: Yes

---

5. Review Comments to the Author

Please use the space provided to explain your answers to the questions above. You may also include additional comments for the author, including concerns about dual publication, research ethics, or publication ethics. (Please upload your review as an attachment if it exceeds 20,000 characters)

Reviewer #1: Dear Editor,
I write to submit my review comments on the manuscript titled "High-dose dexmedetomidine versus dexamethasone supplementation to interscalene brachial plexus block in arthroscopic shoulder surgery: a randomized, controlled trial."

Comments

Abstract: Kindly state the duration of motor and sensory block for the two groups and explicitly state the difference in the duration, confidence interval for the difference in duration. The same must be done for DOA.
Response: We have revised the Abstract (Results) to report the DOA, DOS and DOM for both groups, and we now explicitly present the between-group difference with the corresponding 95% CI for each outcome on **Page 2, Line 27:**
Results: *Dexmedetomidine and dexamethasone had a similar DOA (16.8 vs 11.4 h; median difference in location 5.24 h, 95% CI -0.47 to 9.17; p = 0.112). The dexmedetomidine group had a significantly longer duration of sensory (19.8 vs 17.3 h; mean difference 2.45 h, 95% CI 0.23 to 4.65; p = 0.031) and motor block (19.9 vs 17.8 h; median difference in location 2.23 h, 95% CI 1.02 to 3.60; p = 0.008). Postoperative pain score at 6, 12, 18, and 24 hours, rebound pain, postoperative nausea and vomiting, patient satisfaction, and opioid consumption were comparable. The dexmedetomidine group had a higher incidence of hypotension, bradycardia, and sedation in the post-anesthesia care unit.*

Line 160: Separate statistical analysis from power analysis. There should be a separate section for power analyses.
Response: A separate section for the power analysis has been added on **Page 9, Line 163**, as follows:

Sample Size and Power Calculation

Previous studies reported that groups receiving 5 mg of dexamethasone or dexmedetomidine 2 mcg / kg provided a mean DOA ± standard deviation (SD) of 18 ± 1.95 and 20.4 ± 3.96 hours, respectively [7, 22]. A superiority trial was designed using an automated sample size calculator program, determining a statistical significance at a two-sided level of 0.05 (type I error = 5%) and 80% power (type II error = 20%); each group required a sample size of 50 individuals. Accounting for approximately a 10% dropout rate, each group should consist of 56 individuals, totaling a sample size of 112 individuals.

Major concern: Authors did not include the measurement scale of the outcome measures (continuous, count, discrete, categorical, time to an event, etc) and omitted statistical analyses used in addressing the other objectives regarding the secondary outcome measures. Kindly include in the statistical analyses section how you analyzed the secondary endpoints.
Response: We have revised the Statistical analysis section to clearly define the measurement scale and corresponding analysis for each endpoint on **Page 9 Line 172** as follows:

Statistics or analysis or statistical analyses

Outcome measures were classified as time-to-event, continuous, or categorical variables.

The primary outcome, DOA (hours), was analyzed as a time-to-event variable using Kaplan–Meier survival curves, with between-group comparison by the log-rank test. Median [interquartile range, IQR] DOA and the corresponding median difference in location with 95% confidence intervals (CIs) were reported for descriptive and comparative purposes.

Continuous secondary outcomes included DOS and DOM (hours), intraoperative fentanyl consumption (μg/kg/h), postoperative morphine consumption (mg), NRS pain scores (0–10) at 0, 6, 12, 18, and 24 hours, rebound pain severity (NRS difference), and patient satisfaction scores (0–100). Normality was assessed with the Kolmogorov–Smirnov test. Normally distributed variables were summarized as mean ± standard deviation (SD), and between-group comparisons were performed using the independent-samples t test; group differences were expressed as mean differences (MDs) with 95% CIs derived from IBM SPSS Statistics. Non-normally distributed variables were presented as median (IQR) and compared with the Mann–Whitney U test, and the between-group effect was expressed as the Brown–Mood median difference in location with 95% CIs, obtained using the coin package in R. Longitudinal NRS scores were further evaluated using a regression model including time, treatment group, and their interaction, with adjusted coefficients and 95% CIs reported from marginal estimates.

Categorical secondary outcomes—rebound pain incidence (yes/no), hypotension in the block room, bradycardia in the block room, intraoperative hypotension, intraoperative bradycardia, PONV in the PACU, PONV on the ward, sedation score >0 at 60 minutes (yes/no), and other adverse events—were summarized as frequencies (percentages). Between-group comparisons were made using the Chi-square test or Fisher's exact test, as appropriate, and the effect size was reported as risk differences (RDs) with 95% CIs, estimated in R using the fmsb package.

All tests were two-sided with a significance level of 0.05. Primary and secondary analyses and calculation of MDs with 95% CIs were performed using IBM SPSS Statistics, Version 26.0 (IBM Corp., Armonk, NY, USA). Brown–Mood median differences and risk differences with 95% CIs were obtained using R (R Foundation for Statistical Computing, Vienna, Austria) in RStudio (version 2023.09.1; Posit Software, PBC), with the coin package for the Brown–Mood median test and the fmsb package for risk-difference estimation.

Line 194: Authors stated that “while the median DOA appeared longer in the dexmedetomidine group compared to the dexamethasone group (16.8 [14.2–19.3] hours vs. 11.9 [7.05–16.8] hours), this difference was not statistically significant” but never stated the difference and the corresponding confidence interval

Response: We have revised the Results section on **Page 12, Line 224** to explicitly report the between-group difference in DOA and the corresponding 95% confidence interval, in addition to the p-value as follows.

While the median DOA appeared longer in the dexmedetomidine group compared to the dexamethasone group (16.8 [9.62–19.2] hours vs. 11.4 [5.76–18.1] hours; median difference in location 5.24 hours, 95% CI -0.47 to 9.17; p = 0.112), this difference was not statistically significant.

Authors must be consistent in reporting the results. Sometimes, mean and SD are reported; in other cases, mean and confidence intervals are reported between the two groups.
Table 2 must report the mean or median difference and corresponding 95% CI intervals where appropriate. That must be captured in the third column, and p-values may follow.

Response: we have added group differences along with 95% CI as the third column of Table 2 on **Page 13, Line 243** as follows:

Table 2. Comparison of the duration of analgesic, sensory and motor block, perioperative opioid consumption, patient satisfaction, and adverse effects. (n = 107)

Characteristics	Dexmedetomidine (n = 55)	Dexamethasone (n = 52)	Group difference (95% CI)	p-value
Duration of analgesia (hours)	16.8 [9.62-19.2]	11.4 [5.76-18.1]	5.24 (-0.47 to 9.17)	0.112
Duration of sensory block (hours)	19.8 ± 5.34	17.3 ± 5.64	MD 2.45 (0.23 to 4.65)	0.031
Duration of motor block (hours)	19.9 [16.6-23.1]	17.8 [14.1-19.6]	2.23 (1.02 to 3.60)	0.008
Rebound pain	16 (29.1%)	13 (25.5%)	RD 3.6% (-13.3 to 20.5%)	0.678
Rebound pain severity	6.5 [3.25-8]	5 [4-8]	1 (-4 to 3)	0.965
Intraoperative consumption of fentanyl (mcg / kg / h)	0.56 [0.39-0.78]	0.66 [0.49-0.91]	-0.10 (-0.17 to 0.04)	0.104
Postoperative morphine consumption (mg)	7 [4-13]	5 [2-13]	2 (-1 to 6)	0.324
Patient satisfaction (0-100)	100 [90-100]	100 [90-100]	20 (-0.01 to 20.00)	0.966
Adverse effects				
Hypotension in the block room	2 (3.6%)	2 (3.8%)	RD -0.2% (-7.4 to 7.0%)	1.000
Bradycardia in the block room	13 (23.6%)	3 (5.8%)	RD 17.9% (5.0 to 30.8%)	0.010
Intraoperative hypotension	53 (96.4%)	27 (51.9%)	RD 44.4% (30.0 to 59.9%)	< 0.001
Intraoperative bradycardia	30 (54.5%)	14 (26.9%)	RD 27.6% (9.8 to 45.5%)	0.004
PONV at PACU	1 (1.8%)	0 (0%)	RD 1.8% (-1.7 to 5.3%)	1.000
PONV in the ward	14 (25.5%)	7 (13.5%)	RD 12.0% (-2.8 to 26.8%)	0.118
Sedation score > 0 at 60 minutes	6 (10.9%)	0 (0%)	RD 10.9% (2.7 to 19.1%)	0.027

Values are expressed as medians [interquartile range] or numbers (percentages), except for duration of sensory block, which is expressed as mean ± standard deviation. Group differences are reported as the Brown–Mood median difference in location, mean differences (MDs), or risk differences (RDs), each with corresponding 95% confidence intervals. Sedation score 0 indicates awake and alert; 1 indicates drowsy but easily arousable; 2 indicates somnolent and asleep most of the time; and 3 indicates unarousable or only arousable with significant physical stimulation. Abbreviations: NRS, numerical rating scale; PACU, postanesthetic care unit; PONV, postoperative nausea and vomiting. A p-value < 0.05 was considered statistically significant.

Something must be wrong with the analyses somewhere if you compare the Duration of analgesia (hours), Duration of sensory block (hours), and Duration of motor block (hours). The difference seems to be bigger with the duration of analgesia, but the difference is not statistically significant compared to the other two, where the difference seems to be small but significant. Kindly check the assumptions under the key statistical test that was conducted. Even though this is from RCT, there is no problem fitting models (OLS or quantile regression models where appropriate) that adjust for baseline covariates. May be that will give a clearer picture about the effect size

Response: We thank the reviewer for this careful observation and have re-checked our analyses and assumptions. The three outcomes differ in their statistical structure, which explains the apparent discrepancy between effect size and statistical significance.

First, DOA is a time-to-event endpoint with right-censoring (some patients did not request IV PCA morphine or report pain during admission). Accordingly, DOA was analyzed using Kaplan–Meier curves with log-rank testing (Fig. 2). The censoring and greater dispersion (including a long right tail) reduce precision for DOA and can yield a wider uncertainty around the group contrast despite a numerically larger difference.

In contrast, DOS and DOM are fully observed continuous outcomes without censoring and show tighter distributions, leading to more precise estimates and narrower confidence intervals (**Supplementary Fig. S2).** Therefore, smaller absolute differences in DOS/DOM may still reach statistical significance.

We confirm that assumptions were reviewed: distributional checks for continuous outcomes (using non-parametric methods when appropriate) and appropriate survival methods for censored DOA data. Given the randomized design and balanced baseline characteristics (Table 1), we retained unadjusted analyses as primary; however, we have improved transparency by reporting effect estimates with 95% confidence intervals (Table 2) and by adding Supplementary Fig. S2 to visually display the dispersion of DOA, DOS, and DOM across groups.

Reviewer #2: It would be easier for reader if sedation level was observed with standardized sedation scales like the Richmond or Ramsay scale. Futher studies should include intravenous comparison of dexmedetomidine and dexamethason. Very nice RCT.

Response: We use the short-form Ramsay Sedation Score, which ranges from 0 to 3, because it is widely adopted both in our center and throughout Thailand for patients under anesthesia. We sincerely appreciate your advice and kind compliments.

Reviewer #3: Please can you introduce in introduction section DOA,DOS,DOM, it is hard to follow it latter.
So you introduce intervention, but you can introduce also secondary outcomes.
Also what is rationale to have them all? This is matter of writing introduction, that is more easy for readers to follow methods, results and discussion.
Response: We have revised the Background section to introduce and define the DOA, DOS and DOM on **Page 5, Line 74** as follows:

Because pain, sensory, and motor fibers differ in size and myelination, they have different sensitivities to local anesthetics(23). Peripheral nerve block efficacy is therefore commonly described by the DOA (block placement to first pain or rescue analgesia) and the durations of sensory and motor block (DOS and DOM; block placement to recovery of sensation and motor strength, respectively)(24, 25) to differentiate between prolonged analgesia, sensory and motor block.

Methodology and results section is ok.
Response: -

Discussion: can you find references and improve interpretation of the primary and secondary outcomes.
Response: We have revised the Discussion to provide a clearer, evidence-based interpretation of both the primary and secondary outcomes. Specifically, we (i) incorporated an additional reference on the dose–response plateau of perineural dexmedetomidine and the near-ceiling effect of 4–5 mg dexamethasone, (ii) used these data to explain why 2 µg/kg dexmedetomidine prolonged DOS/DOM but did not improve DOA, and (iii) contextualised the higher rates of bradycardia, hypotension, and sedation with published reports of dose-dependent hemodynamic effects on **Page 15, Line 280** as follows:

In light of prior dose-finding work(26) indicating a dose–response plateau for perineural dexmedetomidine with long-acting local anesthetics (optimal ≈30–50 µg), our 2 µg/kg regimen likely exceeded the range that yields additional analgesic benefit while increasing dose-related hemodynamic effects. Moreover, the comparator dose of dexamethasone (5 mg) is close to the ceiling of its analgesia-prolonging effect, leaving little headroom for superiority. Consistent with these pharmacologic considerations, we observed longer sensory and motor block with dexmedetomidine but no advantage in DOA. This discrepancy is also compatible with our time-to-event definition of DOA (time to first pain or first IV PCA activation, with censoring in patients who never triggered either event) and the fact that interscalene block does not cover all potential nociceptive inputs; thus, prolongation of block characteristics does not necessarily translate into delayed analgesic demand. Collectively, these factors help explain why the primary endpoint did not show superiority despite pharmacological prolongation of DOS and DOM and a numerically longer DOA.

The concluding paragraph was also updated to reflect that, taken together with the literature, high-dose dexmedetomidine does not offer DOA superiority over dexamethasone while increasing adverse effects on **Page 18, Line 341** as follows:

In conclusion, this study compared 2 µg/kg of dexmedetomidine and 5 mg of dexamethasone as adjuvants to bupivacaine in interscalene brachial plexus block for shoulder surgery. While the median DOA appeared longer in the dexmedetomidine group, the difference in this time-to-event outcome was not statistically significant. Despite dexmedetomidine producing more hemodynamic and sedative effects than dexamethasone, these were manageable and transient. Given its longer sensory and motor blockade, dexmedetomidine may be considered a suitable adjuvant for selected, well-monitored patients, whereas dexamethasone remains the safer option in routine practice. These

findings suggest that, at the doses studied, both adjuvants are viable options when used with bupivacaine, although the lack of DOA superiority combined with a higher adverse-effect burden argues against routine use of high-dose dexmedetomidine over dexamethasone in this setting.

Dexmedatomidine side effect versus dexmetason. Can you improve conclusions?
Response: We appreciate the reviewer's attention to this important issue regarding side effects. We have revised the conclusion to highlight the adverse effects associated with dexmedetomidine and to include our team's perspective on their clinical relevance on **Page 18, Line 344**, as shown in the previous response.

Despite dexmedetomidine producing more hemodynamic and sedative effects than dexamethasone, these were manageable and transient. Given its longer sensory and motor blockade, dexmedetomidine may be considered a suitable adjuvant for selected, well-monitored patients, whereas dexamethasone remains the safer option in routine practice.

Reviewer #4: Dear Authors,

I would recommend that you reformulate section 39-44 or to remove it completely. Yes, pain is a mayor symptom we want to address but in my opinion this section is unnecessary.
Response: We agree and have streamlined the Background section. The original first paragraph has been removed, and the key information on pain severity has been integrated into the following paragraph on **page 4, line 41**, which now reads:

Interscalene brachial plexus block (ISB) targeting cervical nerve roots C5 to C7 is often performed to provide safe and effective postoperative analgesia in shoulder surgery, where postoperative pain is commonly moderate to severe(1). Continuous peripheral nerve block catheters offer the advantage of prolonged analgesia. However, improper execution of this highly skilled technique, combined with an unprepared postoperative care team, can result to catheter dislodgement and suboptimal analgesia. As an alternative, single-shot ISB is a simpler approach that provides effective analgesia for a limited duration.

Line 57: one might think that dexamethasone exhibits its antiemetic function by immunosuppression. While it is anti-inflammatory, it is not key mechanism in antiemesis. I recommend to change it.
Response: We agree and have removed the statement on dexamethasone's effect on PONV, as this is not central to our study. The paragraph on **page 4, line 48**, has been revised to read:

To prolong the duration of postoperative analgesia, long-acting local anesthetics are often used in conjunction with adjuvants. Dexamethasone, a corticosteroid, has consistently been shown in systematic reviews to significantly prolong the duration of analgesia (DOA) when used with local anesthetics(2, 3). It exerts analgesic effects by inhibiting phospholipase A2 and stimulating the glucocorticoid system, leading to a systemic anti-inflammatory effect. It is believed that dexamethasone extends the DOA by acting on C fibers, which are responsible for transmitting pain signals. Dexamethasone also inhibits potassium channel function, preventing nerve cells stimulation and the transmission of pain signals to the brain (4). This slows the onset of pain and provides longer pain relief in conjunction with local anesthetics, with a study showing perineural administrations providing longer-lasting effects(5, 6, 7). A systematic review identified 4 mg as a ceiling dose for perineural dexamethasone, extending analgesia by an average of 8 hours when combined with long-acting local anesthetics(8). Despite this, dexamethasone is generally avoided in poorly controlled diabetes due to causing elevated blood glucose.

Line 111: previously taken before. Unnecessary. Also, do you have data from these patients, is it possible these medications interfere with pain scores?
Response: We have removed the redundant phrase "previously taken before" and clarified the sentence to indicate that no additional analgesic premedication was given; only patients' usual chronic analgesic medications, when present, were continued as part of standard care (Methods, page 7, lines 111). We did not systematically collect detailed data on these chronic analgesic regimens. However, because of randomization, any potential influence on postoperative pain scores should be evenly distributed between groups.

Line 131: How many of these patients were excluded and in which groups were they?
Response: We have specified that all ISBs were successful and that no patients were excluded due to failed ISB (Results, page 11 line 207). We additionally specify that patients who had a failed ISB would be withdrew from the study (Material and methods, **page 8, line 133**).

Line 159: What kind of scale is it? Is it standardized?
Response: In Thailand and in our hospital, we usually assess patient satisfaction using a 0–100 scale. While this is not a gold standard method, it is simple for patients to understand and has been widely used in previous practice.

Line 237: I would change the opening of discussion.
I would point out that this higher dose of dexmedetomidine brings significant side effects without any additional benefits compared to dexamethasone.
Response: Thank you for this suggestion. We have revised the opening paragraph of the Discussion to emphasize that the higher dose of dexmedetomidine (2 µg/kg) did not provide additional clinical benefit over dexamethasone (5 mg) in terms of DOA, postoperative opioid use, or pain scores, while it was associated with increased bradycardia and hypotension. The Discussion on **Page 15, Line 268** now begins as follows:

Discussion

In this double-blind randomized trial, a relatively high dose of perineural dexmedetomidine (2 µg/kg) used as an interscalene block adjuvant produced longer sensory and motor block but was clearly associated with higher rates of sedation, bradycardia, and hypotension. Importantly, this escalation in dose did not confer any additional clinical benefit over dexamethasone 5 mg, as DOA, postoperative opioid consumption, pain scores, PONV, and patient satisfaction remained comparable between groups.

---

6. PLOS authors have the option to publish the peer review history of their article (what does this mean?). If published, this will include your full peer review and any attached files.

If you choose "no", your identity will remain anonymous but your review may still be made public.

Do you want your identity to be public for this peer review? For information about this choice, including consent withdrawal, please see our Privacy Policy.
Reviewer #1: No
Reviewer #2: No
Reviewer #3: No
Reviewer #4: No
