# Supplementary material for: High-dose dexmedetomidine versus dexamethasone supplementation to interscalene brachial plexus block in arthroscopic shoulder surgery: A randomized, controlled trial
Source: PLoS One. 2026 Aug 7;21(8):e0353121. doi: 10.1371/journal.pone.0353121 (PMC13450720; doi:10.1371/journal.pone.0353121)
Supplement: Supplementary file 7 [file pone.0353121.s008.pdf]

Manuscript ID: PONE-D-25-27662
Title: High-dose dexmedetomidine versus dexamethasone supplementation to interscalene brachial plexus block in arthroscopic shoulder surgery: a randomized, controlled trial
Journal: PLOS ONE
Corresponding author: Suppachai Poolsuppasit

Reviewers' comments:

Reviewer's Responses to Questions

Comments to the Author

1. If the authors have adequately addressed your comments raised in a previous round of review and you feel that this manuscript is now acceptable for publication, you may indicate that here to bypass the “Comments to the Author” section, enter your conflict of interest statement in the “Confidential to Editor” section, and submit your "Accept" recommendation.

Reviewer #1: All comments have been addressed

Reviewer #3: All comments have been addressed

Reviewer #4: All comments have been addressed

2. Is the manuscript technically sound, and do the data support the conclusions?

The manuscript must describe a technically sound piece of scientific research with data that supports the conclusions. Experiments must have been conducted rigorously, with appropriate controls, replication, and sample sizes. The conclusions must be drawn appropriately based on the data presented.

Reviewer #1: Yes

Reviewer #3: Yes

Reviewer #4: Yes

3. Has the statistical analysis been performed appropriately and rigorously?

Reviewer #1: Yes

Reviewer #3: Yes

Reviewer #4: I Don't Know

4. Have the authors made all data underlying the findings in their manuscript fully available?

The PLOS Data policy requires authors to make all data underlying the findings described in their manuscript fully available without restriction, with rare exception (please refer to the Data Availability Statement in the manuscript PDF file). The data should be provided as part of the manuscript or its supporting information, or deposited to a public repository. For example, in addition to summary statistics, the data points behind means, medians and variance measures

should be available. If there are restrictions on publicly sharing data—e.g. participant privacy or use of data from a third party—those must be specified.

Reviewer #1: Yes

Reviewer #3: Yes

Reviewer #4: Yes

5. Is the manuscript presented in an intelligible fashion and written in standard English?

PLOS ONE does not copyedit accepted manuscripts, so the language in submitted articles must be clear, correct, and unambiguous. Any typographical or grammatical errors should be corrected at revision, so please note any specific errors here.

Reviewer #1: Yes

Reviewer #3: No

Reviewer #4: Yes

6. Review Comments to the Author

Please use the space provided to explain your answers to the questions above. You may also include additional comments for the author, including concerns about dual publication, research ethics, or publication ethics. (Please upload your review as an attachment if it exceeds 20,000 characters)

Reviewer #1: All comments related to statistical analyses have been addressed to my satisfaction including the results section.

Reviewer #3: English revision would be strongly recommended in summary, introduction and discussion.
Response: We have revised the English language throughout the Introduction (Background), Discussion and Summary (Conclusion) to improve grammar, clarity, and readability.

In the conclusion, if two techniques are not statistically significant, then it it irrelevant what is dexmedatomidine group having higher score, it is not evidence that dexmedatomidine is better.

Please write 3-4 strong sentence that are exact and without disscusion.

Example:
This study conclude there was no difference in DOA between groups. (All groups had clinically acceptable valuesm of DOA) ...
They are not different regarding secondary outcomes, except sedation levels...
Response: We have revised the conclusion to avoid implying superiority where the primary outcome was not statistically different on Page 18 Line 342 as follows:
In conclusion, this randomized trial found no statistically significant difference in the DOA between dexmedetomidine (2 µg/kg) and dexamethasone (5 mg) as adjuvants to bupivacaine for interscalene block in arthroscopic shoulder surgery. Dexmedetomidine produced longer DOS and DOM compared with dexamethasone. Perioperative bradycardia, intraoperative hypotension, and early postoperative

sedation occurred more frequently with dexmedetomidine; however, postoperative pain scores and opioid consumption were similar between groups.

Reviewer #4: All my comments were addressed appropriately. Both the introduction and discussion are now more appropriately formatted.

7. PLOS authors have the option to publish the peer review history of their article (what does this mean?). If published, this will include your full peer review and any attached files.

If you choose “no”, your identity will remain anonymous but your review may still be made public.

Do you want your identity to be public for this peer review? For information about this choice, including consent withdrawal, please see our Privacy Policy.

Reviewer #1: No

Reviewer #3: No

Reviewer #4: No
