# Supplementary material for: High-dose dexmedetomidine versus dexamethasone supplementation to interscalene brachial plexus block in arthroscopic shoulder surgery: A randomized, controlled trial
Source: PLoS One. 2026 Aug 7;21(8):e0353121. doi: 10.1371/journal.pone.0353121 (PMC13450720; doi:10.1371/journal.pone.0353121)
Supplement: Supplementary file 8 [file pone.0353121.s009.pdf]

Manuscript ID: PONE-D-25-27662
Title: High-dose dexmedetomidine versus dexamethasone supplementation to interscalene brachial plexus block in arthroscopic shoulder surgery: a randomized, controlled trial
Journal: PLOS ONE
Corresponding author: Suppachai Poolsuppasit

Journal Requirements:
If the reviewer comments include a recommendation to cite specific previously published works, please review and evaluate these publications to determine whether they are relevant and should be cited. There is no requirement to cite these works unless the editor has indicated otherwise.
Response: Noted

[Note: HTML markup is below. Please do not edit.]

Reviewers' comments:

Reviewer's Responses to Questions
Comments to the Author

1. If the authors have adequately addressed your comments raised in a previous round of review and you feel that this manuscript is now acceptable for publication, you may indicate that here to bypass the "Comments to the Author" section, enter your conflict of interest statement in the "Confidential to Editor" section, and submit your "Accept" recommendation.
Reviewer #1: All comments have been addressed
Reviewer #3: All comments have been addressed
Reviewer #5: (No Response)

---

2. Is the manuscript technically sound, and do the data support the conclusions?

The manuscript must describe a technically sound piece of scientific research with data that supports the conclusions. Experiments must have been conducted rigorously, with appropriate controls, replication, and sample sizes. The conclusions must be drawn appropriately based on the data presented.
Reviewer #1: Yes
Reviewer #3: Yes
Reviewer #5: (No Response)

---

3. Has the statistical analysis been performed appropriately and rigorously?
Reviewer #1: Yes
Reviewer #3: Yes
Reviewer #5: (No Response)

---

4. Have the authors made all data underlying the findings in their manuscript fully available?

The PLOS Data policy requires authors to make all data underlying the findings described in their manuscript fully available without restriction, with rare exception (please refer to the Data Availability Statement in the manuscript PDF file). The data should be provided as part of the manuscript or its supporting information, or deposited to a public repository. For example, in addition to summary statistics, the data points behind means, medians and variance measures should be available. If there are restrictions on publicly sharing data—e.g. participant privacy or use of data from a third party—those must be specified.
Reviewer #1: Yes
Reviewer #3: Yes
Reviewer #5: (No Response)

---

5. Is the manuscript presented in an intelligible fashion and written in standard English?

PLOS ONE does not copyedit accepted manuscripts, so the language in submitted articles must be clear, correct, and unambiguous. Any typographical or grammatical errors should be corrected at revision, so please note any specific errors here.
Reviewer #1: Yes
Reviewer #3: Yes
Reviewer #5: (No Response)

---

6. Review Comments to the Author

Please use the space provided to explain your answers to the questions above. You may also include additional comments for the author, including concerns about dual publication, research ethics, or publication ethics. (Please upload your review as an attachment if it exceeds 20,000 characters)

Reviewer #1: The authors have comprehensively addressed all the comments and concerns raised in my previous review of the manuscript
Response: We thank Reviewer #1 for confirming that all concerns have been addressed.

Reviewer #3: All comments are addressed. Please just correct flow of sentence in conclusion.

1. Dexamethasone group produced longer DOS and DOM compared with dexamethasone group.
Response: we have corrected the sentence as suggested.

2. The postoperative pain scores and opioid consumption was similar between groups.
Response: we have corrected the sentence as suggested.

3. Postoperative bradycardia, intraoperative hypotension ......were similar between groups..

Response: We respectfully note that these events were not similar between groups. Table 2 shows that bradycardia and hypotension were significantly more frequent in the dexmedetomidine group: block room bradycardia (23.6% vs 5.8%, $p = 0.010$), intraoperative hypotension (96.4% vs 51.9%, $p < 0.001$), and intraoperative bradycardia (54.5% vs 26.9%, $p = 0.004$). We have revised the conclusion accordingly (Page 21, Line 501):
"Although dexmedetomidine significantly prolonged sensory and motor block duration, this did not translate into a meaningful clinical benefit and was associated with higher rates of hemodynamic instability and sedation."

Reviewer #5: This randomized controlled trial addresses a clinically relevant question and offers a novel comparison between high-dose dexmedetomidine and dexamethasone as adjuvants to interscalene block. The study is well-structured; however, several important issues should be addressed:

Major Points
1. Primary Outcome Interpretation
Despite a notable numerical difference in duration of analgesia (~5 hours), statistical significance was not achieved. This discrepancy should be clarified by discussing clinical versus statistical significance and specifying whether the study was powered for superiority, equivalence, or non-inferiority.
Response: This study was designed and powered as a superiority trial. We have revised the Discussion to clarify that the absence of statistical significance should not be interpreted as evidence of equivalence or non-inferiority. Although the dexmedetomidine group showed a numerically longer median DOA (16.77 vs 12.48 h), the confidence interval included no difference, indicating uncertainty regarding the true benefit. The conclusion now states that dexmedetomidine was not shown to be superior to dexamethasone for prolonging DOA.

Revised Discussion (Page 18, Line 365):
"Although the dexmedetomidine group showed a numerically longer median DOA (16.77 vs 12.48 h), this difference did not reach significance. As this study was designed as a superiority trial, this finding should not be interpreted as evidence of equivalence. The observed difference of approximately 5 hours may be clinically relevant, but the confidence interval included zero, indicating uncertainty regarding the true magnitude of benefit."

Revised Conclusion (Page 21, Line 501):
"In this randomized controlled trial, high-dose perineural dexmedetomidine (2 µg/kg) was not shown to be superior to dexamethasone (5 mg) in prolonging the DOA when used as an adjuvant to bupivacaine for ISB in arthroscopic shoulder surgery."

2. Sample Size and Power
The assumed effect size differs from the observed outcome. A brief post hoc power analysis and discussion of potential underestimation of variability are recommended.
Response: The sample size was based on published data (references 7 and 22) assuming SD 3.96 h, whereas the observed SD was 7.33 h—substantially greater than anticipated. A post hoc calculation

(mean DOA 12.85 vs 14.71 h; pooled SD 7.33; $\alpha = 0.05$ one-sided; $\beta = 0.2$; $\delta = 0.5$) indicated that 358 patients per group (716 total) would be required for 80% power—approximately 6.7 times our enrolled sample. The study was therefore underpowered for the primary outcome. This has been acknowledged in the Discussion.

Revised Discussion (Page 18, Line 370):
"Moreover, a post hoc sample size calculation based on the observed means and variability (mean DOA 12.85 vs 14.71 h; pooled SD 7.33) indicated that 358 patients per group would be required to achieve 80% power, suggesting that the study was substantially underpowered for the primary outcome."

3. Dexmedetomidine Dose Justification
The use of 2 µg/kg appears higher than the commonly recommended fixed doses. The rationale for weight-based dosing should be strengthened, with clearer discussion of dose-dependent risks versus benefits.
Response: We have strengthened the dose rationale in both the Introduction and Discussion. Prior comparative studies used lower doses (50–100 µg or 0.75–1 µg/kg), whereas a dose-finding ISB study (reference 22) found that 2 µg/kg produced the longest DOA but with more intraoperative hypotension. Dosing was based on ideal body weight to standardize exposure. In the Discussion, we now note that although 2 µg/kg prolonged DOS and DOM, it was not superior for DOA and was associated with more adverse hemodynamic events. We also cite evidence (reference 26) of a dose–response plateau at approximately 30–50 µg, suggesting our regimen exceeded the range of additional analgesic benefit.

Revised Introduction (Page 5, Line 78):
"Recent meta-analyses comparing these two adjuvants have reported conflicting findings, with some suggesting that dexamethasone is equivalent or superior to dexmedetomidine in prolonging analgesia (18, 19, 20, 21). Notably, dexmedetomidine doses in these studies ranged from 50–100 µg or 0.75–1 µg/kg. A prior dose-finding study of ISB for arthroscopic shoulder surgery compared perineural dexmedetomidine at 1, 1.5, and 2 µg/kg and found that 2 µg/kg produced the longest DOA, although it was also associated with a higher incidence of intraoperative hypotension (22)."

Revised Introduction (Page 5, Line 91):
"As no study had compared 2 µg/kg perineural dexmedetomidine with 5 mg perineural dexamethasone when combined with bupivacaine, this trial aimed to address this gap. We selected 2 µg/kg dexmedetomidine, calculated from ideal body weight, based on the dose-finding evidence above (22), to standardize drug exposure across patients and to evaluate whether this higher dose—beyond the range previously compared with dexamethasone—would provide additional clinical benefit. However, because this dose exceeds those typically studied, the potential for prolonged analgesia needed to be weighed against the risk of dose-dependent adverse effects, including bradycardia, hypotension, and sedation."

Revised Discussion (Page 18, Line 360):
"Several meta-analyses have indicated that dexamethasone is either superior or equivalent to dexmedetomidine in prolonging the DOA, using dexmedetomidine doses ranging from 50–100 µg or

0.75–1 µg/kg (18, 19, 20, 21). Although 2 µg/kg dexmedetomidine was selected based on prior dose-finding work in arthroscopic shoulder surgery (22), this regimen is higher than the fixed doses commonly used in previous trials.”

Revised Discussion (Page 19, Line 379):
“Prior dose-finding work (26) has identified a dose–response plateau for perineural dexmedetomidine with long-acting local anesthetics at approximately 30–50 µg, suggesting that our 2 µg/kg regimen likely exceeded the range that yields additional analgesic benefit while increasing dose-related hemodynamic effects. Moreover, the comparator dose of dexamethasone (5 mg) approaches the ceiling of its analgesia-prolonging effect, leaving little room for superiority.”

4. Outcome Definition (DOA)
The composite definition (first pain or PCA activation) may introduce bias due to patient-dependent factors. This limitation should be acknowledged.
Response: We have added this as a limitation in the Discussion, noting that the composite DOA definition may have been influenced by individual pain perception, reporting behavior, and willingness to activate PCA, potentially adding variability to the primary outcome.

Revised Discussion (Page 21, Line 487):
“Second, the composite definition of DOA as time to first pain report or first IV PCA activation may have been influenced by patient-dependent factors, including individual pain perception, reporting behavior, and willingness to activate PCA.”

5. Post-Randomisation Exclusions
Excluding patients after randomisation raises concerns regarding adherence to the intention-to-treat principle. Please clarify the analysis approach and consider including ITT-based results.
Response: We acknowledge that analyses were not based on a strict ITT principle. Patients who received non-study analgesics on the day of surgery were excluded because this deviation would confound all pain-related outcomes. Patients requiring conversion to open surgery were withdrawn per prespecified criteria. The number of exclusions remained within the 10% dropout allowance in the original sample size calculation. We have clarified this approach in the Methods, Results, and Discussion.

Revised Methods (Page 11, Line 255):
“The reported analyses were performed in the evaluable population after exclusion of patients with major post-randomisation protocol deviations considered likely to confound pain-related outcomes, specifically receipt of non-study analgesics on the day of surgery. Patients who required conversion to open arthrotomy were withdrawn according to the prespecified withdrawal criteria.”

Revised Results (Page 12, Line 279):
“Three additional patients in the dexamethasone group and one in the dexmedetomidine group were excluded from all analyses because postoperative administration of non-study analgesics on the day of surgery was considered likely to confound postoperative pain-related outcomes.”

Revised Discussion (Page 21, Line 489):
"Third, the reported analyses were not based on a strict intention-to-treat principle, because patients with major post-randomisation protocol deviations were excluded from all analyses; however, these deviations were considered likely to invalidate interpretation of the pain-related outcomes."

6. Statistical Methods
Some methods (e.g., Brown–Mood test) appear unnecessarily complex. Simplification or clearer justification, along with inclusion of clinically interpretable effect sizes, is advised.
Response: Brown–Mood estimates were used to provide clinically interpretable between-group differences in medians with 95% CIs for non-normally distributed outcomes, rather than reporting only p-values. We have clarified this rationale in the Methods and consistently reported effect sizes (median differences in location, mean differences, and risk differences with 95% CIs) throughout the Results.

Revised Statistical Analysis (Page 11, Line 242):
"Non-normally distributed variables were presented as median (IQR) and compared with the Mann–Whitney U test. To provide a clinically interpretable estimate of effect size, the between-group difference was additionally expressed as the Brown–Mood median difference in location with 95% CIs, obtained using the coin package in R."

Revised Results (Page 15, Line 301):
"Dexmedetomidine, compared to dexamethasone, was associated with significantly longer DOS (MD 2.45 h, 95% CI 0.23 to 4.65; p = 0.031) and DOM (median difference in location 2.23 h, 95% CI 1.02 to 3.60; p = 0.008) (Table 2)."

Revised Results (Page 15, Line 307):
"Bradycardia was more frequent in the dexmedetomidine group both in the block room (RD 17.9%, 95% CI 5.0% to 30.8%; p = 0.010) and intraoperatively (RD 27.6%, 95% CI 9.8% to 45.5%; p = 0.004). Intraoperative hypotension was also more frequent with dexmedetomidine (RD 44.4%, 95% CI 30.0% to 59.9%; p < 0.001)."

7. Hemodynamic Adverse Events
The reported rates of hypotension and bradycardia are notably high. Definitions, monitoring protocols, and management strategies should be clearly described, along with a discussion of clinical safety implications.
Response: We have clarified the definitions (hypotension: SBP <80% baseline; bradycardia: HR <50 bpm), monitoring protocol (continuous ECG, NIBP at 5-minute intervals), and management in the Methods. In the Results, we specify that one dexmedetomidine patient required continuous IV dopamine; remaining patients received bolus ephedrine, atropine, or norepinephrine as indicated. In the Discussion, we note that these events were transient and manageable, with no persistent hemodynamic instability in the PACU and no ICU admissions.

Revised Methods (Page 9, Line 190):
"Hypotension was defined as SBP less than 80% of baseline, and bradycardia was defined as heart rate less than 50 beats per minute. Heart rate was monitored continuously via ECG, and blood pressure was measured by NIBP at 5-minute intervals throughout the block room and intraoperative periods. Management of hypotension and bradycardia was at the discretion of the attending anesthesiologist."

Revised Results (Page 15, Line 310):
"One patient in the dexmedetomidine group required intraoperative continuous IV dopamine; the remaining patients received bolus ephedrine, atropine, or norepinephrine as clinically indicated. No patients experienced hypotension or bradycardia in the PACU or required ICU admission."

Revised Discussion (Page 20, Line 434):
"Although bradycardia and hypotension were more frequent in the dexmedetomidine group, these events were transient and manageable, as no patients had persistent hemodynamic instability in the PACU or required ICU admission."

8. Data Availability Statement
Revise to comply with journal policy by stating that all relevant data are included within the manuscript and supporting files.
Response: Revised (Page 30, Line 820): *"All relevant data are within the manuscript and its Supporting Information files. Individual-level de-identified data are available from the corresponding author upon reasonable request, subject to approval by the Siriraj Institutional Review Board, as the data contain potentially sensitive patient information."*

Minor Points:
• Correct typographical and grammatical errors.
Response: All changes are visible as tracked changes in the revised manuscript.

• Ensure consistency in units and statistical reporting formats.
Response: Reviewed and standardized throughout. Effect sizes are consistently reported as mean differences (MDs), Brown–Mood median differences in location, or risk differences (RDs), all with 95% CIs. Units are consistent across the abstract, text, and tables.

• Improve clarity and early definition of abbreviations.
Response: All abbreviations are now defined at first use in both the abstract and main text.

• Revise tables (e.g., unclear satisfaction difference) and ensure complete labelling.
Response: Table 2 column heading changed from "Group difference (95% CI)" to "Effect estimate (95% CI)." A footnote clarifying that median differences in location are not necessarily equal to arithmetic differences between displayed medians has been added. Abbreviations and outcome labels reviewed for completeness.

• Enhance figure clarity (e.g., Kaplan–Meier details).
Response: All figures reviewed and improved for clarity.

• Shorten the AI disclosure section to align with journal standards.
Response: Shortened (Page 31, Line 848):
"Use of artificial intelligence: ChatGPT (OpenAI) and Claude (Anthropic) were used only for language editing and proofreading. All authors reviewed and approved the final manuscript."

Suggested Conclusion (Revised)
High-dose perineural dexmedetomidine (2 µg/kg) did not significantly prolong analgesia compared with dexamethasone (5 mg) as an adjuvant to bupivacaine in an interscalene block. Although it increased sensory and motor block duration, this did not translate into meaningful clinical benefit and was associated with higher rates of hemodynamic instability and sedation. These findings suggest limited advantage and potential safety concerns with higher dosing.
Response: We adopted the reviewer's suggestion with one modification: consistent with the superiority design, we state that dexmedetomidine "was not shown to be superior to" rather than "did not significantly prolong."

Revised Conclusion (Page 21, Line 501):
"In this randomized controlled trial, high-dose perineural dexmedetomidine (2 µg/kg) was not shown to be superior to dexamethasone (5 mg) in prolonging the DOA when used as an adjuvant to bupivacaine for ISB in arthroscopic shoulder surgery. Although dexmedetomidine significantly prolonged sensory and motor block duration, this did not translate into a meaningful clinical benefit and was associated with higher rates of hemodynamic instability and sedation. These findings suggest limited advantage and potential safety concerns with higher dexmedetomidine doses in this clinical setting."

---

7. PLOS authors have the option to publish the peer review history of their article (what does this mean?). If published, this will include your full peer review and any attached files.

If you choose "no", your identity will remain anonymous but your review may still be made public.

Do you want your identity to be public for this peer review? For information about this choice, including consent withdrawal, please see our Privacy Policy.
Reviewer #1: No
Reviewer #3: No
Reviewer #5: **Yes:** Professor Ali Najeh Al-Awwady
