# Supplementary material for: High-dose dexmedetomidine versus dexamethasone supplementation to interscalene brachial plexus block in arthroscopic shoulder surgery: A randomized, controlled trial
Source: PLoS One. 2026 Aug 7;21(8):e0353121. doi: 10.1371/journal.pone.0353121 (PMC13450720; doi:10.1371/journal.pone.0353121)
Supplement: Peer review history

# Peer review: Decision Letter 0

**DOI:** 10.1371/journal.pone.0353121.r001

**Authors:** Benjamin Benzon

29 Oct 2025

PONE-D-25-27662

High-dose dexmedetomidine versus dexamethasone supplementation to interscalene brachial plexus block in arthroscopic shoulder surgery: a randomized, controlled trial

PLOS ONE

Dear Dr. poolsuppasit,

Thank you for submitting your manuscript to PLOS ONE. After careful consideration, we feel that it has merit but does not fully meet PLOS ONE’s publication criteria as it currently stands. Therefore, we invite you to submit a revised version of the manuscript that addresses the points raised during the review process.

**Dear authors, first of all please accept apologies for taking this long to find reviewers. Four reviewers made some specific comments, some are regarding the data analysis, please try to address all of the concerns in logical and coherent manner.**

**I am looking forward to your reply and revised manuscript.**

Please submit your revised manuscript by Dec 13 2025 11:59PM. If you will need more time than this to complete your revisions, please reply to this message or contact the journal office at plosone@plos.org. When you're ready to submit your revision, log on to https://www.editorialmanager.com/pone/ and select the 'Submissions Needing Revision' folder to locate your manuscript file.

Please include the following items when submitting your revised manuscript:

- A rebuttal letter that responds to each point raised by the academic editor and reviewer(s). You should upload this letter as a separate file labeled 'Response to Reviewers'.
- A marked-up copy of your manuscript that highlights changes made to the original version. You should upload this as a separate file labeled 'Revised Manuscript with Track Changes'.
- An unmarked version of your revised paper without tracked changes. You should upload this as a separate file labeled 'Manuscript'.

If you would like to make changes to your financial disclosure, please include your updated statement in your cover letter. Guidelines for resubmitting your figure files are available below the reviewer comments at the end of this letter.

If applicable, we recommend that you deposit your laboratory protocols in protocols.io to enhance the reproducibility of your results. Protocols.io assigns your protocol its own identifier (DOI) so that it can be cited independently in the future. For instructions see: https://journals.plos.org/plosone/s/submission-guidelines#loc-laboratory-protocols. Additionally, PLOS ONE offers an option for publishing peer-reviewed Lab Protocol articles, which describe protocols hosted on protocols.io. Read more information on sharing protocols at https://plos.org/protocols?utm_medium=editorial-email&utm_source=authorletters&utm_campaign=protocols.

We look forward to receiving your revised manuscript.

Kind regards,

Benjamin Benzon, Ph.D., M.D.

Academic Editor

PLOS ONE

**Journal Requirements:**

1. When submitting your revision, we need you to address these additional requirements.

Please ensure that your manuscript meets PLOS ONE's style requirements, including those for file naming. The PLOS ONE style templates can be found at

https://journals.plos.org/plosone/s/file?id=wjVg/PLOSOne_formatting_sample_main_body.pdf and

https://journals.plos.org/plosone/s/file?id=ba62/PLOSOne_formatting_sample_title_authors_affiliations.pdf

2. We note that the grant information you provided in the ‘Funding Information’ and ‘Financial Disclosure’ sections do not match.

When you resubmit, please ensure that you provide the correct grant numbers for the awards you received for your study in the ‘Funding Information’ section.

3. Thank you for stating in your Funding Statement:

The study was supported by Siriraj Research Development Fund (managed by Routine to Research [R2R]), Faculty of Medicine Siriraj Hospital, Grant number is R016332026. The funders had no role in study design, data collection and analysis, decision to publish, or preparation of the manuscript.

Please provide an amended statement that declares *all* the funding or sources of support (whether external or internal to your organization) received during this study, as detailed online in our guide for authors at http://journals.plos.org/plosone/s/submit-now.  Please also include the statement “There was no additional external funding received for this study.” in your updated Funding Statement.

Please include your amended Funding Statement within your cover letter. We will change the online submission form on your behalf.

4. In the online submission form, you indicated that “The data from this study are available from the corresponding author upon reasonable request.”

All PLOS journals now require all data underlying the findings described in their manuscript to be freely available to other researchers, either a. In a public repository, b. Within the manuscript itself, or c. Uploaded as supplementary information.

This policy applies to all data except where public deposition would breach compliance with the protocol approved by your research ethics board. If your data cannot be made publicly available for ethical or legal reasons (e.g., public availability would compromise patient privacy), please explain your reasons on resubmission and your exemption request will be escalated for approval.

5. If the reviewer comments include a recommendation to cite specific previously published works, please review and evaluate these publications to determine whether they are relevant and should be cited. There is no requirement to cite these works unless the editor has indicated otherwise.

[Note: HTML markup is below. Please do not edit.]

Reviewers' comments:

Reviewer's Responses to Questions

**Comments to the Author**

1. Is the manuscript technically sound, and do the data support the conclusions?

The manuscript must describe a technically sound piece of scientific research with data that supports the conclusions. Experiments must have been conducted rigorously, with appropriate controls, replication, and sample sizes. The conclusions must be drawn appropriately based on the data presented.

Reviewer #1: Yes

Reviewer #2: Yes

Reviewer #3: Yes

Reviewer #4: Partly

**********

2. Has the statistical analysis been performed appropriately and rigorously?

Reviewer #1: Yes

Reviewer #2: Yes

Reviewer #3: Yes

Reviewer #4: I Don't Know

**********

3. Have the authors made all data underlying the findings in their manuscript fully available?

The PLOS Data policy requires authors to make all data underlying the findings described in their manuscript fully available without restriction, with rare exception (please refer to the Data Availability Statement in the manuscript PDF file). The data should be provided as part of the manuscript or its supporting information, or deposited to a public repository. For example, in addition to summary statistics, the data points behind means, medians and variance measures should be available. If there are restrictions on publicly sharing data—e.g. participant privacy or use of data from a third party—those must be specified.

Reviewer #1: Yes

Reviewer #2: Yes

Reviewer #3: Yes

Reviewer #4: Yes

**********

4. Is the manuscript presented in an intelligible fashion and written in standard English?

PLOS ONE does not copyedit accepted manuscripts, so the language in submitted articles must be clear, correct, and unambiguous. Any typographical or grammatical errors should be corrected at revision, so please note any specific errors here.

Reviewer #1: Yes

Reviewer #2: Yes

Reviewer #3: No

Reviewer #4: Yes

**********

5. Review Comments to the Author

Please use the space provided to explain your answers to the questions above. You may also include additional comments for the author, including concerns about dual publication, research ethics, or publication ethics. (Please upload your review as an attachment if it exceeds 20,000 characters)

**Reviewer #1:**  Dear Editor,

I write to submit my review comments on the manuscript titled “High-dose dexmedetomidine versus dexamethasone supplementation to interscalene brachial plexus block in arthroscopic shoulder surgery: a randomized, controlled trial.”

Comments

Abstract: Kindly state the duration of motor and sensory block for the two groups and explicitly state the difference in the duration, confidence interval for the difference in duration. The same must be done for DOA.

Line 160: Separate statistical analysis from power analysis. There should be a separate section for power analyses.

Major concern: Authors did not include the measurement scale of the outcome measures (continuous, count, discrete, categorical, time to an event, etc) and omitted statistical analyses used in addressing the other objectives regarding the secondary outcome measures. Kindly include in the statistical analyses section how you analyzed the secondary endpoints.

Line 194: Authors stated that “while the median DOA appeared longer in the dexmedetomidine group compared to the dexamethasone group (16.8 [14.2–19.3] hours vs. 11.9 [7.05–16.8] hours), this difference was not statistically significant” but never stated the difference and the corresponding confidence interval

Authors must be consistent in reporting the results. Sometimes, mean and SD are reported; in other cases, mean and confidence intervals are reported between the two groups.

Table 2 must report the mean or median difference and corresponding 95% CI intervals where appropriate. That must be captured in the third column, and p-values may follow.

Something must be wrong with the analyses somewhere if you compare the Duration of analgesia (hours), Duration of sensory block (hours), and Duration of motor block (hours). The difference seems to be bigger with the duration of analgesia, but the difference is not statistically significant compared to the other two, where the difference seems to be small but significant. Kindly check the assumptions under the key statistical test that was conducted. Even though this is from RCT, there is no problem fitting models (OLS or quantile regression models where appropriate) that adjust for baseline covariates. May be that will give a clearer picture about the effect size

**Reviewer #2:**  It would be easier for reader if sedation level was observed with standardized sedation scales like the Richmond or Ramsay scale. Futher studies should include intravenous comparison of dexmedetomidine and dexamethason. Very nice RCT.

**Reviewer #3:**  Please can you introduce in introduction section DOA,DOS,DOM, it is hard to follow it latter.

So you introduce intervention, but you can introduce also secondary outcomes.

Also what is rationale to have them all? This is matter of writing introduction, that is more easy for readers to follow methods, results and discussion.

Methodology and results section is ok.

Discussion: can you find references and improve interpretation of the primary and secondary outcomes.

Dexmedatomidine side effect versus dexmetason. Can you improve conclusions?

**Reviewer #4:**  Dear Authors,

I would recommend that you reformulate section 39-44 or to remove it completely. Yes, pain is a mayor symptom we want to address but in my opinion this section is unnecessary.

Line 57: one might think that dexamethasone exhibits its antiemetic function by immunosuppression. While it is anti-inflammatory, it is not key mechanism in antiemesis. I recommend to change it.

Line 111: previously taken before. Unnecessary. Also, do you have data from these patients, is it possible these medications interfere with pain scores?

Line 131: How many of these patients were excluded and in which groups were they?

Line 159: What kind of scale is it? Is it standardized?

Line 237: I would change the opening of discussion.

I would point out that this higher dose of dexmedetomidine brings significant side effects without any additional benefits compared to dexamethasone.

**********

6. PLOS authors have the option to publish the peer review history of their article (what does this mean?). If published, this will include your full peer review and any attached files.

If you choose “no”, your identity will remain anonymous but your review may still be made public.

**Do you want your identity to be public for this peer review?**  For information about this choice, including consent withdrawal, please see our Privacy Policy.

Reviewer #1: No

Reviewer #2: No

Reviewer #3: No

Reviewer #4: No

**********

[NOTE: If reviewer comments were submitted as an attachment file, they will be attached to this email and accessible via the submission site. Please log into your account, locate the manuscript record, and check for the action link "View Attachments". If this link does not appear, there are no attachment files.]

To ensure your figures meet our technical requirements, please review our figure guidelines: https://journals.plos.org/plosone/s/figures

You may also use PLOS’s free figure tool, NAAS, to help you prepare publication quality figures: https://journals.plos.org/plosone/s/figures#loc-tools-for-figure-preparation.

NAAS will assess whether your figures meet our technical requirements by comparing each figure against our figure specifications.

# Author comment: Author response to Decision Letter 1

**DOI:** 10.1371/journal.pone.0353121.r002

11 Dec 2025

Please find the attached Response to Reviewers document.

Attachment Submitted filename: Response to reviewer - 8.12.2025.pdf

# Peer review: Decision Letter 1

**DOI:** 10.1371/journal.pone.0353121.r003

**Authors:** Benjamin Benzon

9 Jan 2026

PONE-D-25-27662R1

High-dose dexmedetomidine versus dexamethasone supplementation to interscalene brachial plexus block in arthroscopic shoulder surgery: a randomized, controlled trial

PLOS One

Dear Dr. poolsuppasit,

Thank you for submitting your manuscript to PLOS ONE. After careful consideration, we feel that it has merit but does not fully meet PLOS ONE’s publication criteria as it currently stands. Therefore, we invite you to submit a revised version of the manuscript that addresses the points raised during the review process.

Please submit your revised manuscript by Feb 23 2026 11:59PM. If you will need more time than this to complete your revisions, please reply to this message or contact the journal office at plosone@plos.org. When you're ready to submit your revision, log on to https://www.editorialmanager.com/pone/ and select the 'Submissions Needing Revision' folder to locate your manuscript file.

Please include the following items when submitting your revised manuscript:

- A letter that responds to each point raised by the academic editor and reviewer(s). You should upload this letter as a separate file labeled 'Response to Reviewers'.
- A marked-up copy of your manuscript that highlights changes made to the original version. You should upload this as a separate file labeled 'Revised Manuscript with Track Changes'.
- An unmarked version of your revised paper without tracked changes. You should upload this as a separate file labeled 'Manuscript'.

-->If you would like to make changes to your financial disclosure, please include your updated statement in your cover letter. Guidelines for resubmitting your figure files are available below the reviewer comments at the end of this letter.

If applicable, we recommend that you deposit your laboratory protocols in protocols.io to enhance the reproducibility of your results. Protocols.io assigns your protocol its own identifier (DOI) so that it can be cited independently in the future. For instructions see: https://journals.plos.org/plosone/s/submission-guidelines#loc-laboratory-protocols. Additionally, PLOS ONE offers an option for publishing peer-reviewed Lab Protocol articles, which describe protocols hosted on protocols.io. Read more information on sharing protocols at https://plos.org/protocols?utm_medium=editorial-email&utm_source=authorletters&utm_campaign=protocols.

We look forward to receiving your revised manuscript.

Kind regards,

Benjamin Benzon, Ph.D., M.D.

Academic Editor

PLOS One

Journal Requirements:

If the reviewer comments include a recommendation to cite specific previously published works, please review and evaluate these publications to determine whether they are relevant and should be cited. There is no requirement to cite these works unless the editor has indicated otherwise.

Please review your reference list to ensure that it is complete and correct. If you have cited papers that have been retracted, please include the rationale for doing so in the manuscript text, or remove these references and replace them with relevant current references. Any changes to the reference list should be mentioned in the rebuttal letter that accompanies your revised manuscript. If you need to cite a retracted article, indicate the article’s retracted status in the References list and also include a citation and full reference for the retraction notice.

**Additional Editor Comments:**

Please address these minor issues regrading the language as one reviewer pointed out.

[Note: HTML markup is below. Please do not edit.]

Reviewers' comments:

Reviewer's Responses to Questions

**Comments to the Author**

1. If the authors have adequately addressed your comments raised in a previous round of review and you feel that this manuscript is now acceptable for publication, you may indicate that here to bypass the “Comments to the Author” section, enter your conflict of interest statement in the “Confidential to Editor” section, and submit your "Accept" recommendation.

Reviewer #1: All comments have been addressed

Reviewer #3: All comments have been addressed

Reviewer #4: All comments have been addressed

**********

2. Is the manuscript technically sound, and do the data support the conclusions?

The manuscript must describe a technically sound piece of scientific research with data that supports the conclusions. Experiments must have been conducted rigorously, with appropriate controls, replication, and sample sizes. The conclusions must be drawn appropriately based on the data presented.

Reviewer #1: Yes

Reviewer #3: Yes

Reviewer #4: Yes

**********

3. Has the statistical analysis been performed appropriately and rigorously?

Reviewer #1: Yes

Reviewer #3: Yes

Reviewer #4: I Don't Know

**********

4. Have the authors made all data underlying the findings in their manuscript fully available?

The PLOS Data policy requires authors to make all data underlying the findings described in their manuscript fully available without restriction, with rare exception (please refer to the Data Availability Statement in the manuscript PDF file). The data should be provided as part of the manuscript or its supporting information, or deposited to a public repository. For example, in addition to summary statistics, the data points behind means, medians and variance measures should be available. If there are restrictions on publicly sharing data—e.g. participant privacy or use of data from a third party—those must be specified.

Reviewer #1: Yes

Reviewer #3: Yes

Reviewer #4: Yes

**********

5. Is the manuscript presented in an intelligible fashion and written in standard English?

PLOS ONE does not copyedit accepted manuscripts, so the language in submitted articles must be clear, correct, and unambiguous. Any typographical or grammatical errors should be corrected at revision, so please note any specific errors here.

Reviewer #1: Yes

Reviewer #3: No

Reviewer #4: Yes

**********

6. Review Comments to the Author

Please use the space provided to explain your answers to the questions above. You may also include additional comments for the author, including concerns about dual publication, research ethics, or publication ethics. (Please upload your review as an attachment if it exceeds 20,000 characters)

Reviewer #1: All comments related to statistical analyses have been addressed to my satisfaction including the results section.

Reviewer #3: English revision would be strongly recommended in summary, introduction and discussion.

In the conclusion, if two techniques are not statistically significant, then it it irrelevant what is dexmedatomidine group having higher score, it is not evidence that dexmedatomidine is better.

Please write 3-4 strong sentence that are exact and without disscusion.

Example:

This study conclude there was no difference in DOA between groups. (All groups had clinically acceptable valuesmof DOA) ...

They are not different regarding secondary outcomes, except sedation levels...

Reviewer #4: All my comments were addressed appropriately. Both the introduction and discussion are now more appropriately formatted.

**********

7. PLOS authors have the option to publish the peer review history of their article (what does this mean?). If published, this will include your full peer review and any attached files.

If you choose “no”, your identity will remain anonymous but your review may still be made public.

**Do you want your identity to be public for this peer review?**  For information about this choice, including consent withdrawal, please see our Privacy Policy.

Reviewer #1: No

Reviewer #3: No

Reviewer #4: No

**********

[NOTE: If reviewer comments were submitted as an attachment file, they will be attached to this email and accessible via the submission site. Please log into your account, locate the manuscript record, and check for the action link "View Attachments". If this link does not appear, there are no attachment files.]

To ensure your figures meet our technical requirements, please review our figure guidelines: https://journals.plos.org/plosone/s/figures

You may also use PLOS’s free figure tool, NAAS, to help you prepare publication quality figures: https://journals.plos.org/plosone/s/figures#loc-tools-for-figure-preparation.

NAAS will assess whether your figures meet our technical requirements by comparing each figure against our figure specifications.

# Author comment: Author response to Decision Letter 2

**DOI:** 10.1371/journal.pone.0353121.r004

28 Jan 2026

Please find the details in the attached document

Attachment Submitted filename: Response to reviewer - 27012026.pdf

# Peer review: Decision Letter 2

**DOI:** 10.1371/journal.pone.0353121.r005

**Authors:** Alejandro Torrado Pacheco

30 Mar 2026

PONE-D-25-27662R2

High-dose dexmedetomidine versus dexamethasone supplementation to interscalene brachial plexus block in arthroscopic shoulder surgery: a randomized, controlled trial

PLOS One

Dear Dr. poolsuppasit,

Thank you for submitting your manuscript to PLOS ONE. After careful consideration, we feel that it has merit but does not fully meet PLOS ONE’s publication criteria as it currently stands. Therefore, we invite you to submit a revised version of the manuscript that addresses the points raised during the review process.

An additional reviewer has assessed the manuscript, and requests clarification on certain methodological points. Could you please carefully revise the manuscript to address all comments raised?

Please submit your revised manuscript by May 11 2026 11:59PM. If you will need more time than this to complete your revisions, please reply to this message or contact the journal office at plosone@plos.org. When you're ready to submit your revision, log on to https://www.editorialmanager.com/pone/ and select the 'Submissions Needing Revision' folder to locate your manuscript file.

Please include the following items when submitting your revised manuscript:

- A letter that responds to each point raised by the academic editor and reviewer(s). You should upload this letter as a separate file labeled 'Response to Reviewers'.
- A marked-up copy of your manuscript that highlights changes made to the original version. You should upload this as a separate file labeled 'Revised Manuscript with Track Changes'.
- An unmarked version of your revised paper without tracked changes. You should upload this as a separate file labeled 'Manuscript'.

If you would like to make changes to your financial disclosure, please include your updated statement in your cover letter. Guidelines for resubmitting your figure files are available below the reviewer comments at the end of this letter.

If applicable, we recommend that you deposit your laboratory protocols in protocols.io to enhance the reproducibility of your results. Protocols.io assigns your protocol its own identifier (DOI) so that it can be cited independently in the future. For instructions see: https://journals.plos.org/plosone/s/submission-guidelines#loc-laboratory-protocols. Additionally, PLOS ONE offers an option for publishing peer-reviewed Lab Protocol articles, which describe protocols hosted on protocols.io. Read more information on sharing protocols at https://plos.org/protocols?utm_medium=editorial-email&utm_source=authorletters&utm_campaign=protocols.

As the corresponding author, your ORCID iD is verified in the submission system and will appear in the published article. PLOS supports the use of ORCID, and we encourage all coauthors to register for an ORCID iD and use it as well. Please encourage your coauthors to verify their ORCID iD within the submission system before final acceptance, as unverified ORCID iDs will not appear in the published article. *Only*  the individual author can complete the verification step; PLOS staff *cannot*  verify ORCID iDs on behalf of authors.

We look forward to receiving your revised manuscript.

Kind regards,

Alejandro Torrado Pacheco, PhD

Associate Editor

PLOS One

Journal Requirements:

If the reviewer comments include a recommendation to cite specific previously published works, please review and evaluate these publications to determine whether they are relevant and should be cited. There is no requirement to cite these works unless the editor has indicated otherwise.

[Note: HTML markup is below. Please do not edit.]

Reviewers' comments:

Reviewer's Responses to Questions

**Comments to the Author**

1. If the authors have adequately addressed your comments raised in a previous round of review and you feel that this manuscript is now acceptable for publication, you may indicate that here to bypass the “Comments to the Author” section, enter your conflict of interest statement in the “Confidential to Editor” section, and submit your "Accept" recommendation.

Reviewer #1: All comments have been addressed

Reviewer #3: All comments have been addressed

Reviewer #5: (No Response)

**********

2. Is the manuscript technically sound, and do the data support the conclusions?

The manuscript must describe a technically sound piece of scientific research with data that supports the conclusions. Experiments must have been conducted rigorously, with appropriate controls, replication, and sample sizes. The conclusions must be drawn appropriately based on the data presented.

Reviewer #1: Yes

Reviewer #3: Yes

Reviewer #5: (No Response)

**********

3. Has the statistical analysis been performed appropriately and rigorously?

Reviewer #1: Yes

Reviewer #3: Yes

Reviewer #5: (No Response)

**********

4. Have the authors made all data underlying the findings in their manuscript fully available?

The PLOS Data policy requires authors to make all data underlying the findings described in their manuscript fully available without restriction, with rare exception (please refer to the Data Availability Statement in the manuscript PDF file). The data should be provided as part of the manuscript or its supporting information, or deposited to a public repository. For example, in addition to summary statistics, the data points behind means, medians and variance measures should be available. If there are restrictions on publicly sharing data—e.g. participant privacy or use of data from a third party—those must be specified.

Reviewer #1: Yes

Reviewer #3: Yes

Reviewer #5: (No Response)

**********

5. Is the manuscript presented in an intelligible fashion and written in standard English?

PLOS ONE does not copyedit accepted manuscripts, so the language in submitted articles must be clear, correct, and unambiguous. Any typographical or grammatical errors should be corrected at revision, so please note any specific errors here.

Reviewer #1: Yes

Reviewer #3: Yes

Reviewer #5: (No Response)

**********

6. Review Comments to the Author

Please use the space provided to explain your answers to the questions above. You may also include additional comments for the author, including concerns about dual publication, research ethics, or publication ethics. (Please upload your review as an attachment if it exceeds 20,000 characters)

Reviewer #1: The authors have comprehensively addressed all the comments and concerns raised in my previous review of the manuscript

Reviewer #3: All comments are addressed. Please just correct flow od sentence in conclusion.

1. Dexamethasone group produced longer DOS and DOM compared with dexamethasone group.

2. The postoperative pain scores and opioid consumption was similar between groups.

3. Postoperative bradycardia, intraoperative hypotension ......were similar between groups..

Reviewer #5: This randomized controlled trial addresses a clinically relevant question and offers a novel comparison between high-dose dexmedetomidine and dexamethasone as adjuvants to interscalene block. The study is well-structured; however, several important issues should be addressed:

Major Points

1. Primary Outcome Interpretation

Despite a notable numerical difference in duration of analgesia (~5 hours), statistical significance was not achieved. This discrepancy should be clarified by discussing clinical versus statistical significance and specifying whether the study was powered for superiority, equivalence, or non-inferiority.

2. Sample Size and Power

The assumed effect size differs from the observed outcome. A brief post hoc power analysis and discussion of potential underestimation of variability are recommended.

3. Dexmedetomidine Dose Justification

The use of 2 µg/kg appears higher than the commonly recommended fixed doses. The rationale for weight-based dosing should be strengthened, with clearer discussion of dose-dependent risks versus benefits.

4. Outcome Definition (DOA)

The composite definition (first pain or PCA activation) may introduce bias due to patient-dependent factors. This limitation should be acknowledged.

5. Post-Randomisation Exclusions

Excluding patients after randomisation raises concerns regarding adherence to the intention-to-treat principle. Please clarify the analysis approach and consider including ITT-based results.

6. Statistical Methods

Some methods (e.g., Brown–Mood test) appear unnecessarily complex. Simplification or clearer justification, along with inclusion of clinically interpretable effect sizes, is advised.

7. Hemodynamic Adverse Events

The reported rates of hypotension and bradycardia are notably high. Definitions, monitoring protocols, and management strategies should be clearly described, along with a discussion of clinical safety implications.

8. Data Availability Statement

Revise to comply with journal policy by stating that all relevant data are included within the manuscript and supporting files.

Minor Points:

• Correct typographical and grammatical errors.

• Ensure consistency in units and statistical reporting formats.

• Improve clarity and early definition of abbreviations.

• Revise tables (e.g., unclear satisfaction difference) and ensure complete labelling.

• Enhance figure clarity (e.g., Kaplan–Meier details).

• Shorten the AI disclosure section to align with journal standards.

Suggested Conclusion (Revised)

High-dose perineural dexmedetomidine (2 µg/kg) did not significantly prolong analgesia compared with dexamethasone (5 mg) as an adjuvant to bupivacaine in an interscalene block. Although it increased sensory and motor block duration, this did not translate into meaningful clinical benefit and was associated with higher rates of hemodynamic instability and sedation. These findings suggest limited advantage and potential safety concerns with higher dosing.

**********

7. PLOS authors have the option to publish the peer review history of their article (what does this mean?). If published, this will include your full peer review and any attached files.

If you choose “no”, your identity will remain anonymous but your review may still be made public.

**Do you want your identity to be public for this peer review?**  For information about this choice, including consent withdrawal, please see our Privacy Policy.

Reviewer #1: No

Reviewer #3: No

Reviewer #5: **Yes:**  Professor Ali Najeh Al-Awwady

**********

[NOTE: If reviewer comments were submitted as an attachment file, they will be attached to this email and accessible via the submission site. Please log into your account, locate the manuscript record, and check for the action link "View Attachments". If this link does not appear, there are no attachment files.]

To ensure your figures meet our technical requirements, please review our figure guidelines: https://journals.plos.org/plosone/s/figures

You may also use PLOS’s free figure tool, NAAS, to help you prepare publication quality figures: https://journals.plos.org/plosone/s/figures#loc-tools-for-figure-preparation.

NAAS will assess whether your figures meet our technical requirements by comparing each figure against our figure specifications.

Attachment Submitted filename: Peer Review Report.pdf

# Author comment: Author response to Decision Letter 3

**DOI:** 10.1371/journal.pone.0353121.r006

10 May 2026

I attached file response to reviewer.

Attachment Submitted filename: Response to reviewer - plosone 1052026.pdf

# Peer review: Decision Letter 3

**DOI:** 10.1371/journal.pone.0353121.r007

**Authors:** James Mockridge

19 Jun 2026

High-dose dexmedetomidine versus dexamethasone supplementation to interscalene brachial plexus block in arthroscopic shoulder surgery: a randomized, controlled trial

PONE-D-25-27662R3

Dear Dr. poolsuppasit,

We’re pleased to inform you that your manuscript has been judged scientifically suitable for publication and will be formally accepted for publication once it meets all outstanding technical requirements.

Within one week, you’ll receive an e-mail detailing the required amendments. When these have been addressed, you’ll receive a formal acceptance letter and your manuscript will be scheduled for publication.

An invoice will be generated when your article is formally accepted. Please note, if your institution has a publishing partnership with PLOS and your article meets the relevant criteria, all or part of your publication costs will be covered. Please make sure your user information is up-to-date by logging into Editorial Manager at Editorial Manager® and clicking the ‘Update My Information' link at the top of the page. For questions related to billing, please contact billing support.

If your institution or institutions have a press office, please notify them about your upcoming paper to help maximize its impact. If they’ll be preparing press materials, please inform our press team as soon as possible -- no later than 48 hours after receiving the formal acceptance. Your manuscript will remain under strict press embargo until 2 pm Eastern Time on the date of publication. For more information, please contact onepress@plos.org.

Kind regards,

James Mockridge

Staff Editor

PLOS One

Additional Editor Comments (optional):

Reviewers' comments:

Reviewer's Responses to Questions

**Comments to the Author**

1. If the authors have adequately addressed your comments raised in a previous round of review and you feel that this manuscript is now acceptable for publication, you may indicate that here to bypass the “Comments to the Author” section, enter your conflict of interest statement in the “Confidential to Editor” section, and submit your "Accept" recommendation.

Reviewer #3: All comments have been addressed

Reviewer #5: (No Response)

**********

2. Is the manuscript technically sound, and do the data support the conclusions?

The manuscript must describe a technically sound piece of scientific research with data that supports the conclusions. Experiments must have been conducted rigorously, with appropriate controls, replication, and sample sizes. The conclusions must be drawn appropriately based on the data presented.

Reviewer #3: Yes

Reviewer #5: Partly

**********

3. Has the statistical analysis been performed appropriately and rigorously?

Reviewer #3: Yes

Reviewer #5: Yes

**********

4. Have the authors made all data underlying the findings in their manuscript fully available?

The PLOS Data policy requires authors to make all data underlying the findings described in their manuscript fully available without restriction, with rare exception (please refer to the Data Availability Statement in the manuscript PDF file). The data should be provided as part of the manuscript or its supporting information, or deposited to a public repository. For example, in addition to summary statistics, the data points behind means, medians and variance measures should be available. If there are restrictions on publicly sharing data—e.g. participant privacy or use of data from a third party—those must be specified.

Reviewer #3: Yes

Reviewer #5: Yes

**********

5. Is the manuscript presented in an intelligible fashion and written in standard English?

PLOS ONE does not copyedit accepted manuscripts, so the language in submitted articles must be clear, correct, and unambiguous. Any typographical or grammatical errors should be corrected at revision, so please note any specific errors here.

Reviewer #3: Yes

Reviewer #5: Yes

**********

6. Review Comments to the Author

Please use the space provided to explain your answers to the questions above. You may also include additional comments for the author, including concerns about dual publication, research ethics, or publication ethics. (Please upload your review as an attachment if it exceeds 20,000 characters)

Reviewer #3: English language revision is necessary.

Conclusion can be better written. It is in the end with important impact on a reader.

I

Reviewer #5: This is a well-designed randomized trial comparing high-dose dexmedetomidine (2 mcg/kg) vs dexamethasone (5 mg) as adjuvants to bupivacaine for interscalene brachial plexus block in shoulder arthroscopy. The primary finding—no statistically significant difference in duration of analgesia—is clearly presented and supported by the data. Secondary outcomes, including prolonged motor/sensory block and increased hemodynamic effects with dexmedetomidine, are also appropriately reported.

However, prior to final acceptance, the following must be addressed:

Data availability: The current statement (“available from corresponding author upon reasonable request”) does not comply with PLOS ONE’s policy requiring full data availability without restriction. Please either deposit the anonymized dataset in a public repository (e.g., Figshare, Dryad, OSF) with a DOI, or include the data as Supporting Information files.

Minor typographical error: In the abstract (page 10, line 22), “2 mg/kg” of dexmedetomidine should read “2 mcg/kg” to match the manuscript text and methods. Please correct.

No other major concerns. The statistical analysis is appropriate, the manuscript is intelligible, and the conclusions are consistent with the results.

**********

7. PLOS authors have the option to publish the peer review history of their article (what does this mean?). If published, this will include your full peer review and any attached files.

If you choose “no”, your identity will remain anonymous but your review may still be made public.

**Do you want your identity to be public for this peer review?**  For information about this choice, including consent withdrawal, please see our Privacy Policy.

Reviewer #3: No

Reviewer #5: No

**********

# Editor report: Acceptance letter

**DOI:** 10.1371/journal.pone.0353121.r008

**Authors:** James Mockridge

PONE-D-25-27662R3

PLOS One

Dear Dr. Poolsuppasit,

I'm pleased to inform you that your manuscript has been deemed suitable for publication in PLOS One. Congratulations! Your manuscript is now being handed over to our production team.

At this stage, our production department will prepare your paper for publication. This includes ensuring the following:

* All references, tables, and figures are properly cited

* All relevant supporting information is included in the manuscript submission,

* There are no issues that prevent the paper from being properly typeset

You will receive further instructions from the production team, including instructions on how to review your proof when it is ready. Please keep in mind that we are working through a large volume of accepted articles, so please give us a few days to review your paper and let you know the next and final steps.

Lastly, if your institution or institutions have a press office, please let them know about your upcoming paper now to help maximize its impact. If they'll be preparing press materials, please inform our press team within the next 48 hours. Your manuscript will remain under strict press embargo until 2 pm Eastern Time on the date of publication. For more information, please contact onepress@plos.org.

You will receive an invoice from PLOS for your publication fee after your manuscript has reached the completed accept phase. If you receive an email requesting payment before acceptance or for any other service, this may be a phishing scheme. Learn how to identify phishing emails and protect your accounts at https://explore.plos.org/phishing.

If we can help with anything else, please email us at customercare@plos.org.

Thank you for submitting your work to PLOS ONE and supporting open access.

Kind regards,

PLOS ONE Editorial Office Staff

on behalf of

Dr James Mockridge

Staff Editor

PLOS One
